# “Zero-Strain” NiNb_2_O_6_ Fibers for All-Climate Lithium Storage

**DOI:** 10.1007/s40820-024-01497-z

**Published:** 2024-09-27

**Authors:** Yan Zhao, Qiang Yuan, Liting Yang, Guisheng Liang, Yifeng Cheng, Limin Wu, Chunfu Lin, Renchao Che

**Affiliations:** 1 College of Physics, Donghua University, Shanghai, 201620 People’s Republic of China; 2 School of Materials Science and Engineering, Institute of Materials for Energy and Environment, Qingdao University, Qingdao, 266071 People’s Republic of China; 3 Laboratory of Advanced Materials, Shanghai Key Laboratory of Molecular Catalysis and Innovative Materials, Academy for Engineering & Technology, Fudan University, Shanghai, 200438 People’s Republic of China; 4 Zhejiang Laboratory, Hangzhou, 311100 People’s Republic of China; 5 Inner Mongolia University, Hohhot, 010021 People’s Republic of China

**Keywords:** NiNb_2_O_6_ porous fiber, “Zero-strain” mechanism, Electrochemical property, Harsh-temperature operation, *Operando* characterization

## Abstract

Niobates are promising all-climate Li^+^-storage anode material due to their fast charge transport, large specific capacities, and resistance to electrolyte reaction. However, their moderate unit-cell-volume expansion (generally 5%–10%) during Li^+^ storage causes unsatisfactory long-term cyclability. Here, “zero-strain” NiNb_2_O_6_ fibers are explored as a new anode material with comprehensively good electrochemical properties. During Li^+^ storage, the expansion of electrochemical inactive NiO_6_ octahedra almost fully offsets the shrinkage of active NbO_6_ octahedra through reversible O movement. Such superior volume-accommodation capability of the NiO_6_ layers guarantees the “zero-strain” behavior of NiNb_2_O_6_ in a broad temperature range (0.53%//0.51%//0.74% at 25// − 10//60 °C), leading to the excellent cyclability of the NiNb_2_O_6_ fibers (92.8%//99.2% // 91.1% capacity retention after 1000//2000//1000 cycles at 10C and 25// − 10//60 °C). This NiNb_2_O_6_ material further exhibits a large reversible capacity (300//184//318 mAh g^−1^ at 0.1C and 25// − 10//60 °C) and outstanding rate performance (10 to 0.5C capacity percentage of 64.3%//50.0%//65.4% at 25// − 10//60 °C). Therefore, the NiNb_2_O_6_ fibers are especially suitable for large-capacity, fast-charging, long-life, and all-climate lithium-ion batteries.

**Supplementary Information:**

The online version contains supplementary material available at 10.1007/s40820-024-01497-z.

## Highlights

- “Zero-strain” NiNb_2_O_6_ fibers with nanosized primary particles are explored as an all-climate anode material with comprehensively good Li^+^-storage properties.
- The almost completely opposite volume changes of electrochemical inactive NiO_6_ octahedra and active NbO_6_ octahedra are achieved through reversible O movement, leading to the “zero-strain” behavior of NiNb_2_O_6_ with minor unit-cell-volume change and excellent cyclability in a broad temperature range.
- The gained insight can provide guide for the exploration of high-performance energy-storage materials working at harsh temperatures.

**Supplementary Information:**

The online version contains supplementary material available at 10.1007/s40820-024-01497-z.

## Graphical abstract

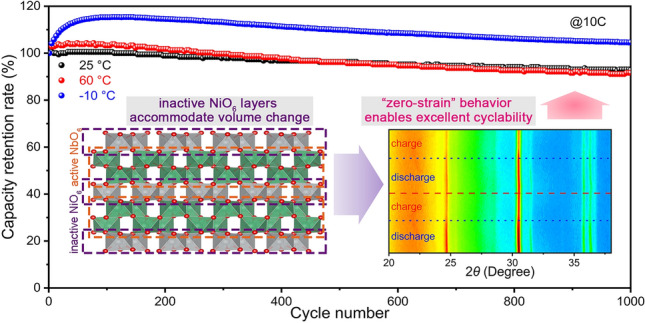

## Introduction

Lithium-ion batteries (LIBs) are very popular electrochemical energy-storage devices. However, the current LIBs still have limitations in terms of energy density, power density, cyclability, safety, and temperature adaptability [1–5]. Especially, both low and high temperatures reduce the energy and power densities of LIBs, rendering them less practical in high altitude/latitude and hot tropical/summer regions [6–9]. Graphite, the most widely-used anode material in current LIBs, possesses a large theoretical capacity (372 mAh g^−1^), excellent electronic conductivity, and low cost, but suffers from a safe problem arising from the easy lithium-dendrite formation [10]. Additionally, high temperatures cause excessive Li^+^ intercalation in graphite, resulting in severe particle cracks and fast capacity decay [11, 12]. At low temperatures, graphite experiences significant increase in charge transfer resistance and notable decrease in Li^+^ diffusivity, which lead to severe rate-performance fade [13, 14]. Li_4_Ti_5_O_12_, another commercial anode material, demonstrates excellent rate capability and cyclability after properly modified [15]. However, Li_4_Ti_5_O_12_ suffers from several issues, including the very limited theoretical capacity (only 175 mAh g^−1^), intensive reaction of Ti^4+^ with electrolyte at high temperatures, and slow Li^+^ diffusivity at low temperatures [15–17].

The recently-developed niobate anode materials provide a viable solution to the aforementioned challenges [12, 18–21]. They possess open crystal structures and multiple lithiation sites with electrochemical-active Nb ions [22–30]. These advantages enable superior charge-transport capability and large-capacity Li^+^ storage over multiple discharge–charge cycles. Additionally, they exhibit resistance to electrolyte reaction, low polarization, and fast ion migration, rendering them suitable for harsh-condition applications [31, 32]. However, their long-term cyclability is unsatisfactory, mainly due to their moderate unit-cell-volume expansion (generally 5%–10%) after lithiation [33]. In this regard, “zero-strain” niobates with minor unit-cell-volume change of < 1% have gained much research interest, which can show minor stress generated during lithiation, thereby avoiding microcrack formation and enabling excellent cyclability [34–36]. Here, zero-strain means no unit-cell-volume change during electrochemical reaction, and the further use of quotes means tiny unit-cell-volume change of < 1%. In 2021, Xia et al. reported NiNb_2_O_6_ submicron particles (averaging at 680 nm) with a “zero-strain” potential through solid-state reaction [37]. This material had high rate performance (10 to 0.5C capacity percentage of 57.4%) and good cyclability (capacity retention of 92.0% after 2500 cycles at 10C) at 25 °C, demonstrating is practicability. To commercialize NiNb_2_O_6_, however, its electrochemical properties need to be further improved, its harsh-temperature operation needs to be investigated, and its “zero-strain” behavior and mechanism need to be clarified.

In this work, we successfully synthesize NiNb_2_O_6_ fibers with primary particles of only 50–100 nm by electrospinning, improving the reversible capacity and rate performance of NiNb_2_O_6_. The maximum unit-cell-volume expansion of NiNb_2_O_6_ is determined for the first time (only + 0.53%// + 0.51%// + 0.74% at 25// − 10//60 °C), and its “zero-strain” behavior in the broad temperature range is identified. During lithiation, the expansion of electrochemical inactive NiO_6_ octahedra almost fully offsets the shrinkage of active NbO_6_ octahedra through reversible O movement, resulting in its “zero-strain” structure stability and excellent cyclability (92.8%//99.2%//91.1% capacity retention after 1000//2000//1000 cycles at 10C and 25// − 10//60 °C). Furthermore, the NiNb_2_O_6_ fibers exhibit a large reversible capacity (300//184//318 mAh g^−1^ at 0.1C and 25// − 10//60 °C) and outstanding rate performance (10 to 0.5C capacity percentage of 64.3%//50.0%//65.4% at 25// − 10//60 °C) in the broad temperature range. Therefore, the good practicability of our modified NiNb_2_O_6_ material in all-climate LIBs is fully revealed.

## Experimental Section

### Material Synthesis

0.02 mol of NbCl_5_ (99.9%, Macklin) was thoroughly mixed with 10 mL of N, N-dimethylformamide (DMF), which was stirred until a translucent orange solution was formed. 0.01 mol of NiCl_2_·6H_2_O (99.9%, Macklin) was added to this solution and stirred until the clear solution turned green in color. Then, 1.0 g of polyvinylpyrrolidone (*M*_w_ = 1,300,000, Macklin) was dissolved in this solution with stirring, forming the electrospinning solution. The electrospinning experiment was conducted on a commercial electrospinning machine (TL-Pro-BM). The electrospinning potential, pumping rate, and distance between the syringe tip and film collector were set to be 16 kV, 0.6 mL h^−1^, and 20 cm, respectively. The electrospun film was heated at 850 °C for 4 h in air, resulting in the formation of NiNb_2_O_6_ fibers.

### Material Characterizations

The crystal structure of NiNb_2_O_6_ was analyzed on an X-ray diffractometer (Rigaku Smart Lab) with Cu K*α* radiation. The General Structure Analysis System (GSAS) Program was employed to Rietveld refine the obtained XRD patterns [38]. Morphology observations were conducted using field emission scanning electron microscopy (FESEM, JEOL JSM-7800F) equipped with energy dispersive spectroscopy (EDS, OXFORD X-Max) and high-resolution transmission electron microscopy (HRTEM, JEOL JEM-2100Plus). The Brunauer–Emmett–Teller (BET) model was used to calculate the specific surface area of the NiNb_2_O_6_ fibers based on the N_2_ adsorption data recorded on an ASAP 2460 surface area analyzer. The Barrett–Joyner–Halenda (BJH) model was employed to compute the pore-size-distribution curve from the desorption branch. The ultraviolet–visible (UV–Vis) absorption spectrum of NiNb_2_O_6_ was recorded using a U-3900H spectrophotometer. The electronic conductivity of NiNb_2_O_6_ was tested by employing a two-probe direct current method on a compacted NiNb_2_O_6_ pellet. The valence changes of NiNb_2_O_6_ on cast electrodes were analyzed using X-ray photoelectron spectroscopy (XPS) on a PHI5000 Versa probe Ш system. In an Ar-filled glove box, the NiNb_2_O_6_ electrodes at different discharge/charge states were disassembled from the coin cells, and then washed with dimethyl carbonate (DMC). After fully dried, the NiNb_2_O_6_ electrodes were sealed in an XPS sample stage before the XPS examinations.

### Electrochemical Characterizations

For the preparation of the working electrodes for half cells, the synthesized NiNb_2_O_6_ powder (active material, 75 wt%), Super-P conductive carbon (for conductivity, 15 wt%), polyvinylidene fluoride (binder, 10 wt%), and N-methyl-2-pyrrolidone (solvent) were mixed in a bottle and stirred for 4 h to form a slurry. The slurry was then cast into copper (Cu) foils with a loading of ~ 1.5 mg cm^−2^, which were fully dried in a vacuum oven at 110 °C for 12 h. The electrochemical tests were conducted on CR2016-type half coin cells assembled in an Ar-filling glove box, with Celgard^®^ 2325 microporous polypropylene films as separators, Li-metal foils as counter electrodes, and 1 M LiPF_6_ in a 1:1:1 volume ratio of ethylene carbonate, diethylene carbonate, and dimethyl carbonate as electrolyte. The galvanostatic intermittent titration technique (GITT) and galvanostatic charge–discharge (GCD) tests within 1.0–3.0 V were performed using a Neware CT-3008 battery tester at different temperatures controlled in an LRHS-101C temperature-variable cryostat system, and cyclic voltammetry (CV) and electrochemical impedance spectroscopy (EIS) experiments were further performed using a Gamry Interface1010E electrochemical workstation.

### *Operando* Characterizations

*Operando* XRD experiments were carried out to study the lattice-parameter variations of NiNb_2_O_6_ during electrochemical reactions at various temperatures of 25, − 10, and 60 °C. The construction process of the in-situ XRD cell at 25 °C followed the same procedure as that of the half cells, except that the NiNb_2_O_6_-based composite film was peeled off from the Cu foil and then coated on a beryllium (Be) plate. This Be plate served as both the X-ray penetration window and current collector within a specially designed module (LIB-XRD, Beijing Scistar Technology). To maintain the desired operating temperature at − 10 or 60 °C, the module (LHTXRD-LN, Beijing Scistar Technology) incorporated an X-ray penetration polyetheretherketone (PEEK) dome and a temperature-control unit [39].

The *operando* TEM test was carried out to track the real-time microstructure changes of the NiNb_2_O_6_ fibers during lithiation and delithiation using a specially-designed electrochemical holder from the X-mech Center in Zhejiang University, which simulated the operation of a half cell [18]. The working electrode was NiNb_2_O_6_ fibers on a W wire, and the counter electrode was Li metal with naturally-formed Li_2_O coating on another W wire. The Li → Li_2_O → NiNb_2_O_6_ lithiation process was observed when the two electrodes were in contact and a proper voltage was applied.

## Results and Discussion

### Structures and Physico-Chemical Characterizations

**Fig. 1:**
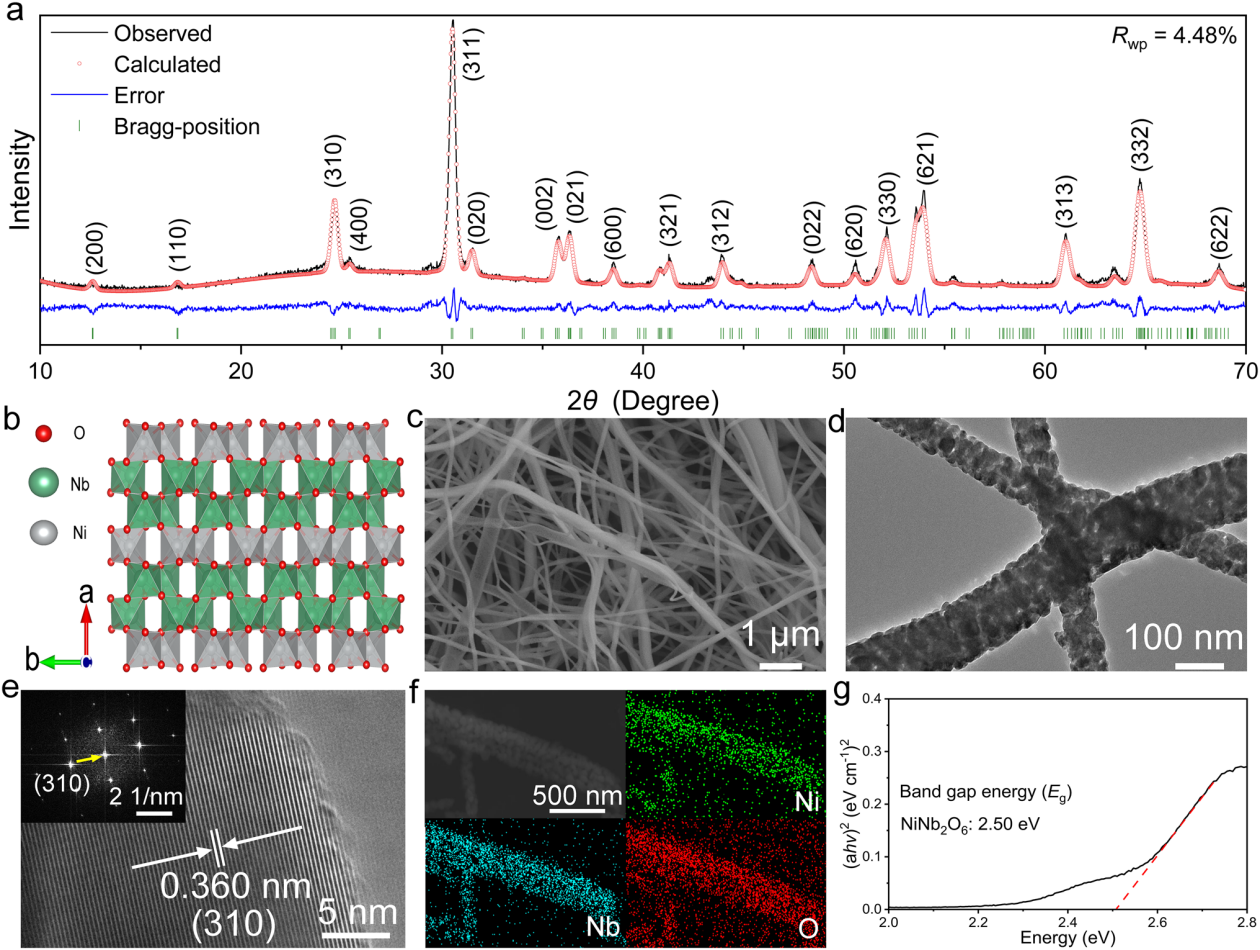
Physico-chemical characterizations of NiNb_2_O_6_ fibers. **a** XRD pattern. **b** Crystal structure viewed along *c* axis. **c** FESEM image. **d** TEM image. **e** HRTEM image (*inset*: FFT). **f** EDX mapping images. **g** Evolution of optical bandgap based on UV–vis absorption spectrum. (Color figure online)

The XRD pattern of the NiNb_2_O_6_ sample is successfully refined by using the Rietveld method with a small residual value (*R*_wp_ = 4.48%, Fig. 1a) [38]. NiNb_2_O_6_ crystallizes in an orthorhombic crystal structure with a *Pbcn* space group. Its main characteristic peaks are located at the Bragg angles of 24.7° and 30.5°, corresponding to its (310) and (311) crystallographic planes (PDF No. 01-072-0481). Its lattice parameters are determined to be *a* = 14.033134(57) Å, *b* = 5.685842(5) Å, *c* = 5.021448(79) Å, and *V* = 400.662(75) Å^3^. In its orthogonal structure, each Ni/Nb ion is surrounded by six O^2−^ ions to form a corner-shared octahedron (Fig. 1b). Along the *a*-axis direction, NiO_6_ and NbO_6_ layers are alternately arranged, forming a stable layered structure with wide channels, which can be conductive to Li^+^ transport and reduce the unit-cell-volume change caused by Li^+^ insertion/extraction. The corner-shared NbO_6_ octahedra and NiO_6_ octahedra are serrated along the *b*-axis direction. Clearly, the main Li^+^ diffusion direction is along the *c*-axis direction [37].

The NiNb_2_O_6_ sample has a morphology of fibers with primary-particle sizes of 50–100 nm and a large BET specific surface area of 8.35 m^2^ g^−1^ (Figs. 1c, d and S1). The hierarchical pore sizes of the NiNb_2_O_6_ fibers are centered at ~ 50 and ~ 20 nm, which correspond to the inter-fiber and inter-particle pores, respectively. The well-defined lattice spacing of 0.360 nm (Fig. 1e) and the corresponding fast Fourier transform (FFT, Fig. 1e *inset*) match with the (310) plane of NiNb_2_O_6_. The homogeneous distributions of Ni, Nb, and O (Fig. 1f) confirm the high purity and uniformity of this NiNb_2_O_6_ material. NiNb_2_O_6_ exhibits a small bandgap of 2.50 eV from its UV–vis absorption spectrum (Fig. 1g), with a tested electronic conductivity reaching 2.2 × 10^−8^ S cm^−1^.

### Li^+^-Storage Properties

**Fig. 2:**
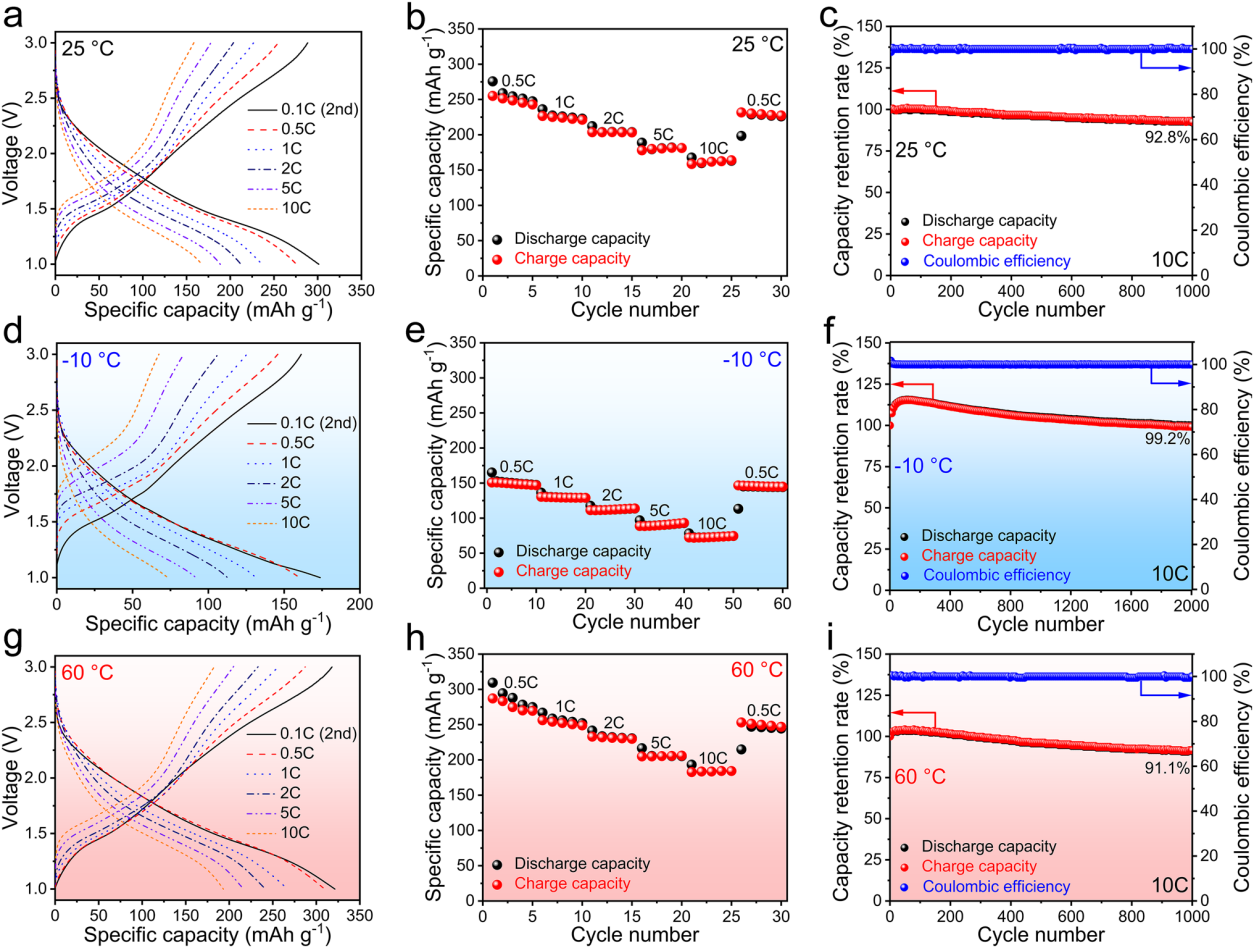
Electrochemical properties of NiNb_2_O_6_ fibers at different temperatures. **a** GCD profiles at different current rates, **b** rate performance, **c** cyclability (after rate-performance test) of NiNb_2_O_6_/Li half-cell at 25 °C, **d** GCD profiles at different current rates, **e** rate performance, **f** cyclability (after rate-performance test) of NiNb_2_O_6_/Li half-cell at − 10 °C, **g** GCD profiles at different current rates, **h** rate performance, **i** cyclability (after rate-performance test) of NiNb_2_O_6_/Li half-cell at 60 °C. (Color figure online)

From the first three-cycle GCD curves of the NiNb_2_O_6_/Li half cell recorded within a safe potential range of 1.0–3.0 V at 0.1C (Fig. S2a), it can be found that the first discharge curve shows a sequence of very fast drop (> 1.7 V) → slow drop (1.7–1.2 V) → plateau (1.2–1.3 V, phase transformation) → fast drop (< 1.2 V), whereas the second and third discharge curves follow a different sequence of very fast drop (> 2.5 V) → slow drop (2.5–1.3 V) → fast drop (< 1.3 V). The charge-curve shapes are almost fully reversible to the discharge-curve ones, except for the first one. This curve discrepancy is primarily attributed to the phase transformation of NiNb_2_O_6_ only occurring during initial lithiation. The NiNb_2_O_6_ fibers deliver a large reversible capacity of 300 mAh g^−1^ in the first cycle, approaching its theoretical capacity (315 mAh g^−1^ based on Nb^5+^  ↔ Nb^3+^). At 0.5C, 1C, 2C, 5C, and 10C, large reversible capacities of 255, 226, 204, 181, and 164 mAh g^−1^ are retained, respectively (Fig. 2a, b). The resulting 10 to 0.5C capacity percentage is as large as 64.3%, which outperforms that of the previously-reported NiNb_2_O_6_ microparticles (57.4%) [37]. The data fully demonstrate the outstanding rate performance of our NiNb_2_O_6_ material. The higher activity of our NiNb_2_O_6_ material is undoubtedly due to its smaller primary particles, which enables larger electrochemical-reaction area. Meanwhile, its cyclability is also outstanding, retaining high capacity retention of 92.8% at 10C after 1000 cycles (Fig. 2c). The Nyquist plots of the NiNb_2_O_6_/Li half cell (Fig. S3) reveal that the charge-transfer resistance decreases with cycling. Such continuous activation also indicates the outstanding cyclability. Furthermore, the LiFePO_4_/NiNb_2_O_6_ full cell also exhibits good electrochemical properties (Fig. S4a, b). Especially, its reversible capacity (212 mAh g^−1^ at 1C) is 100 and 60 mAh g^−1^ larger than that of the LiFePO_4_/T-Nb_2_O_5_ and LiFePO_4_/Li_4_Ti_5_O_12_ full cells, respectively (Fig. S4c–f). These desirable electrochemical properties of the NiNb_2_O_6_ fibers are superior to those of most intercalation-type anode materials previously reported (Table S1).

At − 10 °C, the first discharge curve of NiNb_2_O_6_ significantly differs from that at 25 °C, with the disappearance of the plateau stage (phase transformation) at 1.0–3.0 V (Fig. S2b). The reason for this change might be attributed to the lowered ion motion at low temperatures, which inhibits the phase transformation [40–42]. However, NiNb_2_O_6_ still has a satisfactory reversible capacity of 184 mAh g^−1^ at 0.1C (Fig. S2e), 62% of that at 25 °C. In contrast, the popular Li_4_Ti_5_O_12_ nanoparticles only exhibit a corresponding percentage of only 49% [43]. The rate performance of NiNb_2_O_6_ at − 10 °C retains high, with a 10 to 0.5C capacity percentage of 50.0% (Fig. 2d, e). In addition, the cyclability at − 10 °C is even better than that at 25 °C, exhibiting ultra-high capacity retention of 99.2% at 10C after 2000 cycles (Fig. 2f).

At 60 °C, NiNb_2_O_6_ delivers a 6% larger reversible capacity (318 mAh g^−1^ at 0.1C) and 1.1% larger 10 to 0.5C capacity percentage (65.4%) than those at 25 °C (Figs. S2c and 2g, h), undoubtedly due to the enhanced electrochemical kinetics. Importantly, it still retains high capacity retention of 91.1% at 10C after 1000 cycles (Fig. 2i). In contrast, the corresponding percentages of commercial graphite microparticles and Li_4_Ti_5_O_12_ porous microspheres at the same elevated temperature are respectively only 36.7% after 1000 cycles at 10C and 20.9% after 500 cycles at 5C [12, 44]. To sum up, the NiNb_2_O_6_ fibers have comprehensively good electrochemical properties over a broad temperature range, becoming a practical anode material for all-climate LIBs.

### Electrochemical-Reaction Mechanisms

**Fig. 3:**
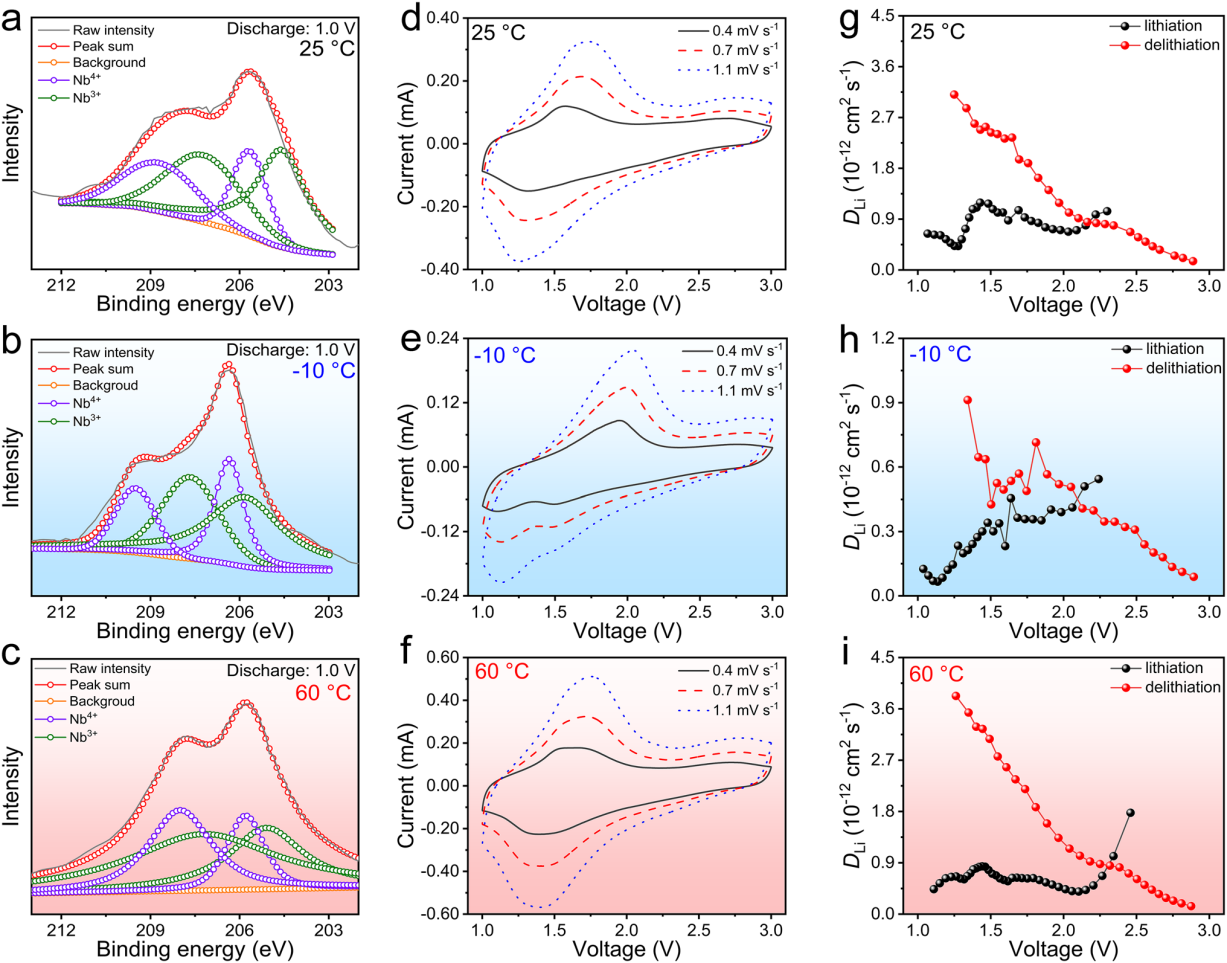
Redox mechanism and electrochemical kinetics of NiNb_2_O_6_ fibers. Ex-situ Nb-3*d* XPS spectra at discharge state (1.0 V): **a** 25, **b** − 10, and **c** 60 °C. CV curves of NiNb_2_O_6_/Li half-cell at different sweep rates: **d** 25, **e** − 10, and **f** 60 °C. Apparent Li^+^ diffusion coefficients during lithiation–delithiation: **g** 25, **h** − 10, and **i** 60 °C. (Color figure online)

The XPS peaks of pristine NiNb_2_O_6_ (Fig. S5d) are located at 210.5 and 207.8 eV, matching with the Nb-3*d*_3/2_ and Nb-3*d*_5/2_ of Nb^5+^, respectively [45, 46]. At the discharge state of 25 °C (1.0 V, Fig. 3a), the peaks at 208.8 and 206.1 eV correspond to Nb^4+^ (33%), and the peaks at 206.9 and 204.2 eV are attributed to Nb^3+^ (67%) [47, 48]. Upon reaching the fully charged state, the valence state of Nb is restored to its original Nb^5+^ configuration (Fig. S5g). Therefore, the active Nb^4+^/Nb^5+^ and Nb^3+^/Nb^4+^ redox couples in NiNb_2_O_6_ are confirmed. The proportion of Nb^3+^ decreases to 20% at − 10 °C, but increases to 74% at 60 °C (Figs. 3b, c; S3e, f, h, i). These two percentage variations can be ascribed to the enhanced electrochemical kinetics at elevated temperatures. In contrast, the binding energies of Ni-2*p*_3/2_ (856.2 eV) and Ni-2*p*_1/2_ (873.8 eV) remain unchanged at different discharged/charged states (Fig. S5a–c) [49], indicating the very stable Ni^2+^ ions during discharge/charge, as expected.

The first-cycle (0.2 mV s^−1^, Fig. S6a) and first four-cycle (0.4 mV s^−1^, Fig. S6d) CV curves of the NiNb_2_O_6_/Li half-cell are displayed at 25 °C. The redox reactions involving Nb^4+^/Nb^5+^ and Nb^3+^/Nb^4+^ couples exhibit weak cathodic // anodic peaks at 1.71//1.77 V and intensive peaks at 1.37//1.49 V, suggesting the fast reduction of Nb^5+^ to Nb^4+^ and a significant amount of Nb^4+^ to Nb^3+^ [19, 45, 46]. These findings match well with the XPS analysis during discharge at 1.0 V. The CV curves show slight peak shifts at large sweeping rates, demonstrating small electrode polarization (Fig. 3d). At − 10 °C, the peak intensities are lower at all the sweep rates, and the peaks become broader with increasing the sweep rate (Figs. 3e and S4e). At 60 °C, the peak intensities observed during the first four cycles at 0.4 mV s^−1^ are ~ 30% higher than that at 25 °C (Fig. S6f), while the results at 0.4–1.1 mV s^−1^ show a similarity to those at 25 °C (Fig. 3f). The increased polarization at the low temperature is mainly ascribed to the reduced electrical conductivity of the active material, decreased rates of ion migration, and constrained capability of charge transfer [19]. Conversely, these issues are not prevalent in high-temperature environments, thereby mitigating the polarization problem.

The Li^+^ diffusivity of the NiNb_2_O_6_ fibers at various temperatures and lithiation/delithiation states is investigated through GITT (Fig. S7) [50]. At 25 °C, the average Li^+^ diffusion coefficients during lithiation and delithiation respectively reach 8.1 × 10^−13^ and 1.4 × 10^−12^ cm^2^ s^−1^ (Fig. 3g). At − 10 °C, the values decrease by only ~ 50% (Fig. 3h), reaching 4.5 × 10^−13^ cm^2^ s^−1^ (lithiation) and 7.7 × 10^−13^ cm^2^ s^−1^ (delithiation). However, larger values of 8.2 × 10^−13^ cm^2^ s^−1^ (lithiation) and 1.8 × 10^−12^ cm^2^ s^−1^ (delithiation) are achieved at 60 °C (Fig. 3i). Such fast Li^+^ diffusivity at different temperatures (Table S2) is verified by the CV tests (Fig. S8) [34], which is undoubtedly attributed to the open and stable layered structure of NiNb_2_O_6_. The temperature increase indeed enhances the electrochemical kinetics, providing a good explanation for the observed variations in the rate performance at 25, − 10, and 60 °C.

At − 10//25//60 °C, the capacitive contribution of the NiNb_2_O_6_ fibers increases with increasing the sweep rates, reaching 94%//84%//82% at 1.1 mV s^−1^ (Fig. S6g–i), which indicates a significant contribution of the capacitive process to the Li^+^ storage [51]. This significant pseudocapacitive behavior can be rooted in the spacious crystal structure that is highly suitable for Li^+^ intercalation, as well as the fiber morphology with a large specific surface area and abundant pores. Since the capacitive process is not limited by solid-state diffusion [52], the capacitive behavior allows for fast charge transport, also benefiting the rate performance of the NiNb_2_O_6_ material [53–58].

### Crystal-Structure Evolutions and “Zero-Strain” Mechanisms

**Fig. 4:**
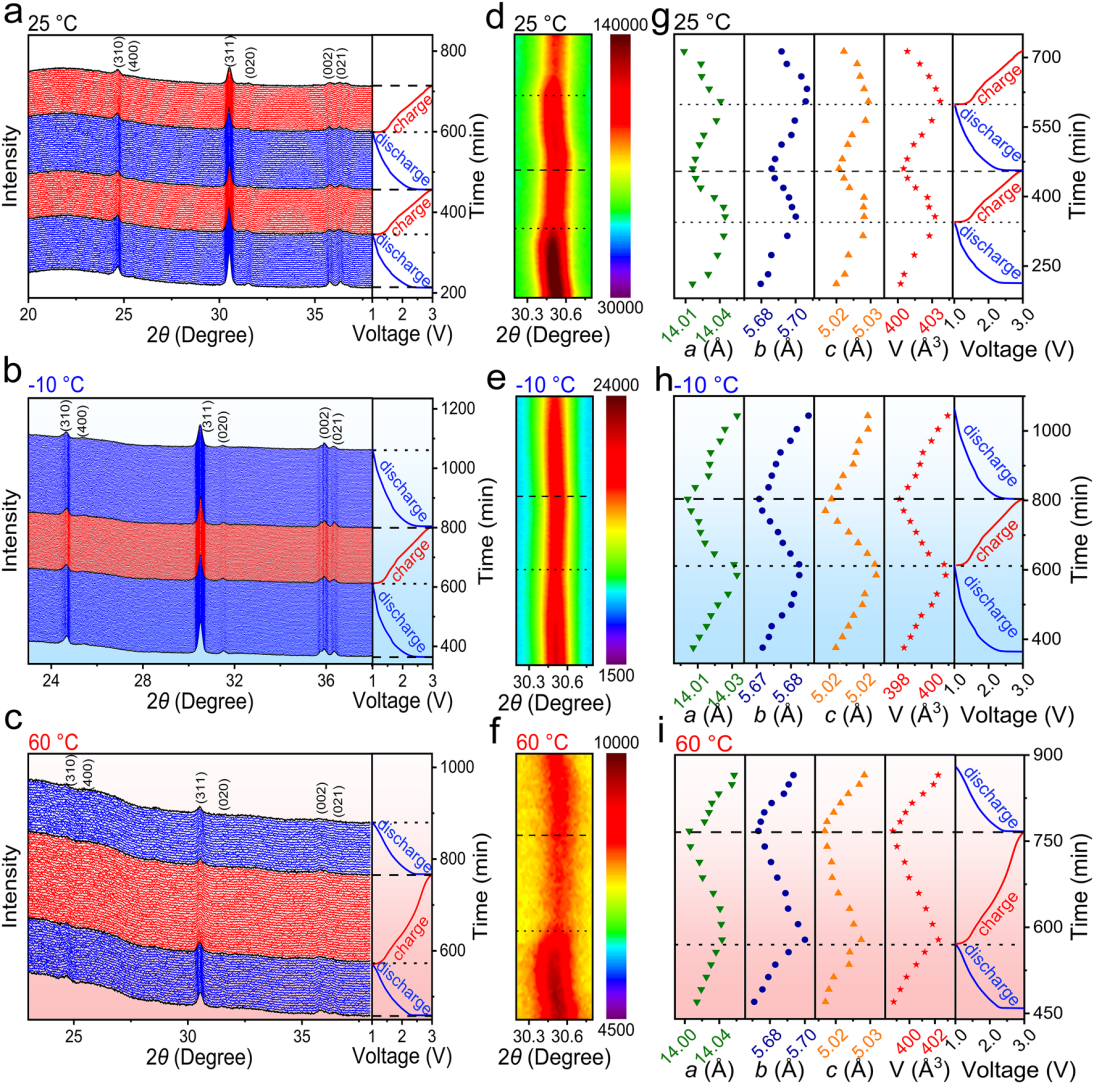
*Operando* XRD characterizations of NiNb_2_O_6_ fibers within 1.0–3.0 V (second discharge to third charge at 0.5C and 25 °C, second discharge to third discharge at 0.1C and − 10 °C, and second discharge to third discharge at 0.5C and 60 °C). *Operando* XRD patterns of NiNb_2_O_6_/Li half-cell with GCD curves: **a** 25, **b** − 10, and **c** 60 °C. 2D *operando* XRD patterns enlarged within 30.3–30.7°: **d** 25, **e** − 10, and **f** 60 °C. Variations in lattice parameters of NiNb_2_O_6_ during discharge–charge: **g** 25, **h** − 10, and **i** 60 °C. (Color figure online)

As can be seen from the first-cycle *operando* XRD patterns (Fig. S9a, d), NiNb_2_O_6_ undergoes a phase transformation when discharge to 1.27 V [18], but the new phase retains the same framework as the initial one. During first lithiation, the diffraction peaks at 24.6, 25.4, 30.4, 31.5, 35.6, and 36.2°, which respectively correspond to the (310), (400), (311), (020), (002), and (021) planes of NiNb_2_O_6_, slightly shift towards lower Bragg angles and then slightly shift towards higher Bragg angles at < 1.25 V (Fig. S10a). During subsequent delithiation, these diffraction peaks consistently shift towards higher Bragg angles, and restore their initial positions. However, during second lithiation without the phase transformation (Fig. 4a, d), all the diffraction peaks consistently shift towards lower Bragg angles, and exhibit the reversible evolution to that during first delithiation, which are highly repeatable during the subsequent cycles (Fig. S11a). Clearly, once the first lithiation process is completed, the crystal structure of NiNb_2_O_6_ becomes very stable.

**Fig. 5:**
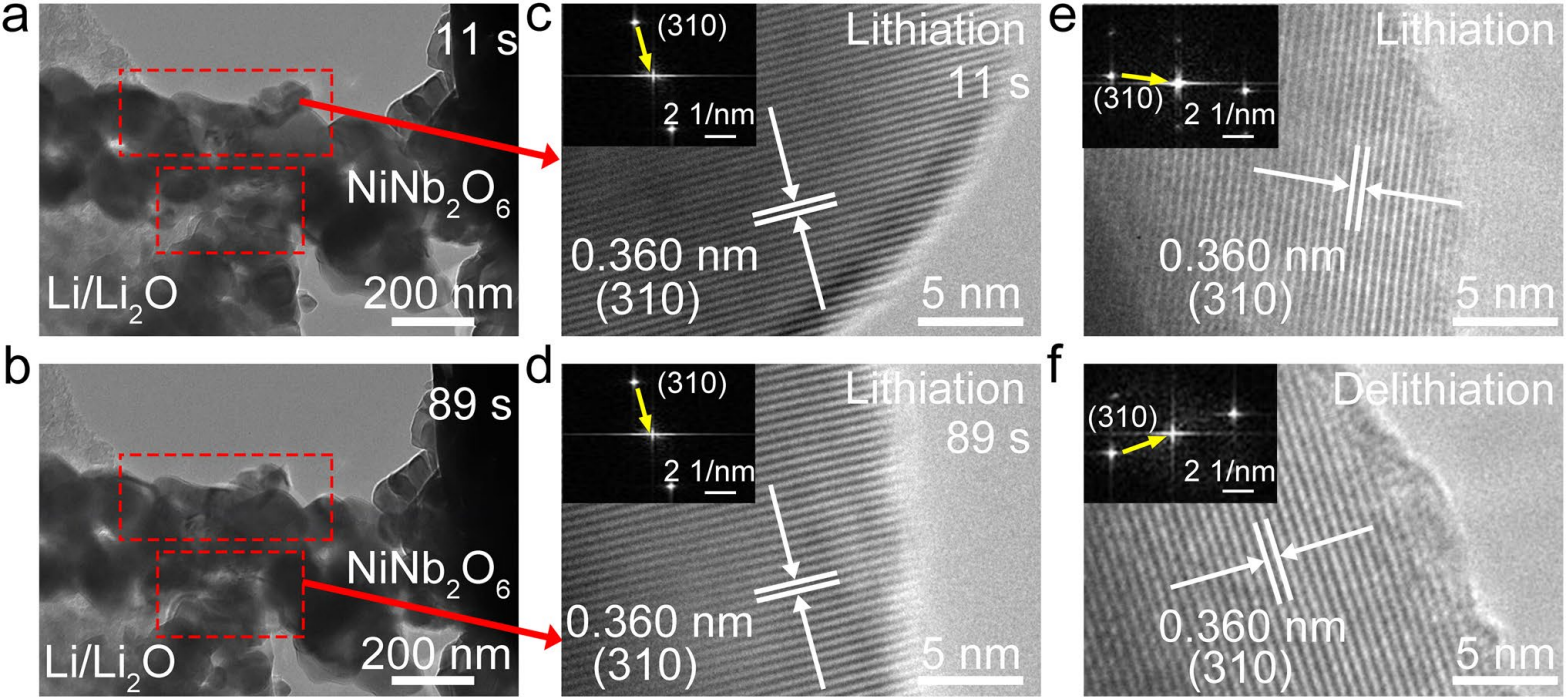
*Operando* and ex-situ TEM examinations of NiNb_2_O_6_. *Operando* TEM images of Li^+^ insertion in NiNb_2_O_6_: **a** 11 and **b** 89 s. *Operando* HRTEM images (*inset*: *operando* FFT) of NiNb_2_O_6_ recorded at lithiation states: **c** 11 and **d** 89 s. Ex-situ HRTEM images (*inset*: ex-situ FFT) of NiNb_2_O_6_: **e** lithiation (1.0 V) and **f** delithiation (3.0 V) states

The lattice-parameter (*a*, *b*, *c*, and *V*) variations of NiNb_2_O_6_ during lithiation–delithiation are determined through Rietveld-refining the *operando* XRD patterns (Figs. 4g, S9g, and S12a). During first lithiation, the *a*, *b*, *c*, and *V* values slowly increase (> 1.25 V) and obviously decrease after the phase transformation (< 1.25 V), and they continue decreasing during first delithiation, which match well with the diffraction-peak shifts. In the subsequent cycles, the lattice-parameter variations are highly reversible (Fig. S11d), slowly increasing during lithiation and then decreasing during delithiation. The total *a-*, *b-*, *c-*, and *V-*value changes after lithiation are determined for the first time, which are as small as + 0.13%, + 0.27%, + 0.13%, and + 0.53%, respectively. During the *operando* TEM test [18], the tested particles exhibit noticeable strain-fringe movement during lithiation, but the variations in the morphology and volume are negligible (Fig. 5a, b; Video S1). In addition, the *operando*/ex-situ HRTEM and FFT characterizations reveal the unchanged (310) lattice spacing at different lithiation/delithiation states (Fig. 5c–f), which are in good agreement with the *operando* XRD characterizations. Importantly, the ex-situ XRD pattern of the NiNb_2_O_6_ electrode after 100 cycles reveals that all the XRD peaks are well maintained (Fig. S13), demonstrating the “zero-strain” characteristic of NiNb_2_O_6_ during long-term cycling.

At − 10 °C, the variations of the *operando* XRD patterns are highly reversible during all the cycles (no phase transformation), and the peak-shift amplitudes are smaller than that at 25 °C (Figs. 4b, e and S9b, e). The maximum *a*-, *b*-, *c*-, and *V*-value changes remain as small as + 0.21%, + 0.23%, + 0.06%, and + 0.51%, respectively (Fig. S9h). In contrast, at 60 °C, the amplitudes of the peak shifts and the variations in the lattice parameters are larger than those at 25 °C (Figs. 4c, f; S9c, f; S10b; and S12b) due to more Li^+^ ions intercalated at the high temperature. The maximum changes of the *a*, *b*, *c*, and *V* values slightly increase to + 0.18%, + 0.44%, + 0.15%, and + 0.74%, respectively (Fig. 4i). Therefore, the “zero-strain” characteristic of NiNb_2_O_6_, with the smallest unit-cell-volume change among the known niobates (Table S3), is clearly identified in a broad temperature range, which well explains the excellent cyclability of our NiNb_2_O_6_ material at various temperatures.

**Fig. 6:**
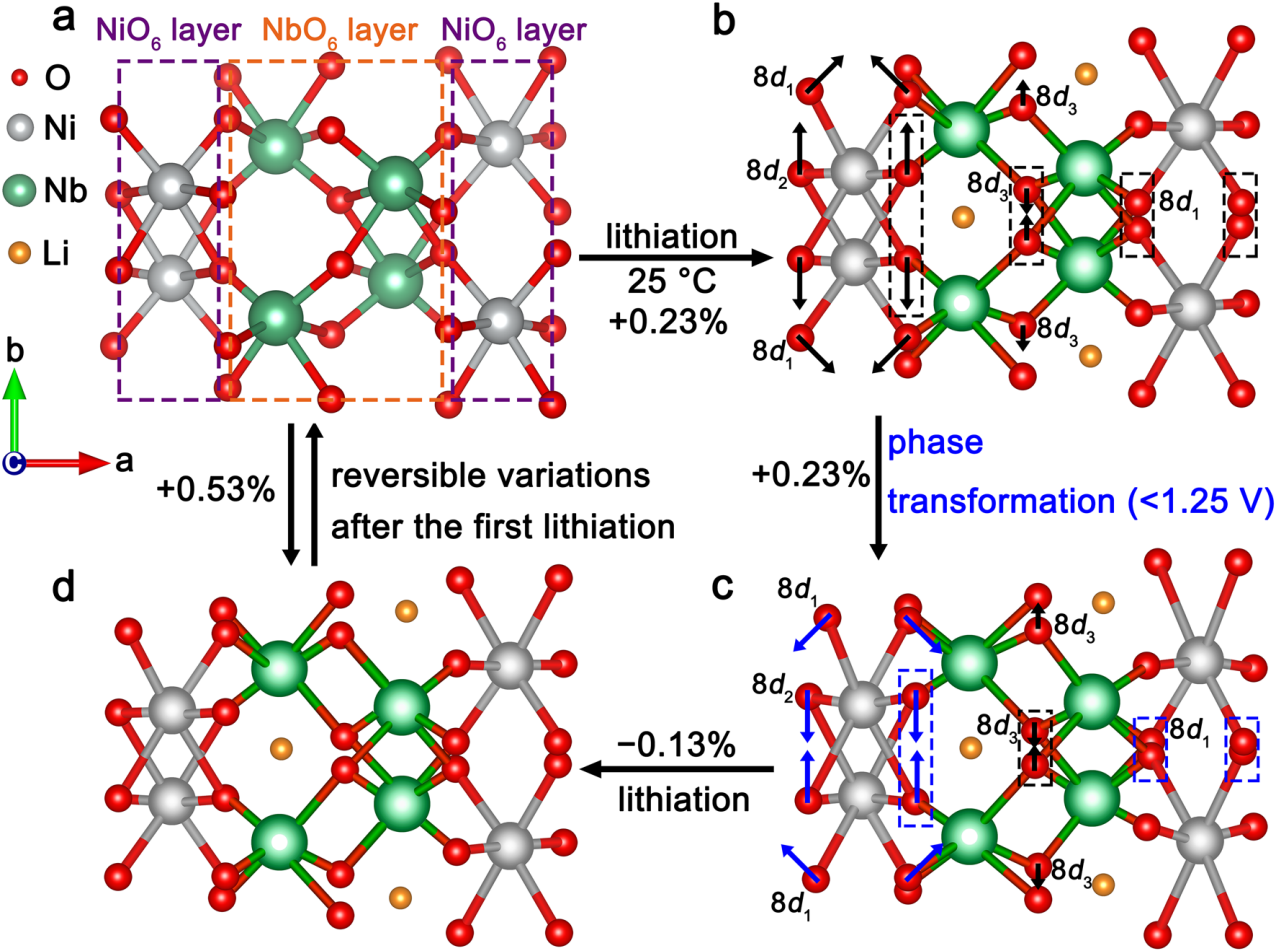
“Zero-strain” mechanism of NiNb_2_O_6_ at 25 °C. Movement of ions from **a** initial stage to **b** partially lithiated stage, then to **c** phase-transformed stage, and finally to **d** final lithiated stage. Li, Ni, Nb, and O are colored by orange, grey, green, and red, respectively. Arrow lengths indicate movement distances. (Color figure online)

The “zero-strain” mechanism of NiNb_2_O_6_ is systematically investigated through *operando* XRD combined with Rietveld refinements. It is found that the movement of the Ni and Nb ions is negligible throughout the electrochemical reaction. During initial lithiation (> 1.3 V) at 25 °C, the adjacent O ions at the 8*d*_1_ (or 8*d*_3_) positions approach each other, while the adjacent O ions at the 8*d*_2_ position move away from each other (Fig. 6a, b and Tables S5, S6), resulting in longer M–O (8*d*_1_) bonds, longer M–O (8*d*_3_) bonds, shorter M–O (8*d*_2_) bonds (M represents Ni or Nb, Table S9), and a 0.23% expansion of the unit-cell volume. During the phase transformation (~ 1.27 V), the three types of O ions suddenly move, but the M–O bonds maintain their initial change trends and the unit-cell volume further expands by 0.23% (Fig. 6b). After the phase transformation (< 1.25 V), the O^2−^ (8*d*_1_) and O^2−^ (8*d*_2_) ions move to the directions opposite to their initial ones (Fig. 6c), leading to shorter M–O (8*d*_1_) bonds, longer M–O (8*d*_2_) bonds, and a unit-cell-volume shrinkage (− 0.13%, Fig. 6d and Tables S7, S8). At 1.0 V, the NiO_6_ octahedra expand by 4.93%, while the NbO_6_ octahedra shrink by 2.23% (Table S10). During first delithiation, the adjacent O (8*d*_1_ or 8*d*_3_) ions move away from each other, while the adjacent O (8*d*_2_) ions approach each other, gradually recovering the initial state (Fig. 6a, d). During subsequent lithiation // delithiation, and the movement of O ion, M–O bond lengths, and polyhedron-volume changes exhibit reversible//identical variations compared to those during first delithiation. At − 10 °C, no sudden ion movement (no phase transformation) is observed, and the ion movement amplitudes and the M–O bond changes are smaller (Fig. S14 and Tables S12–S14). Consequently, the volume changes of the NiO_6_ (+ 2.74%) and NbO_6_ (− 0.83%) octahedra are also smaller (Table S15). In contrast, at 60 °C, the ion movement amplitudes and the M–O bond changes become larger, causing larger NiO_6_ (+ 6.21%) and NbO_6_ (− 3.05%) octahedron-volume changes (Fig. S15 and Tables S17–S22). At all these three temperatures, the volume-expansion percentages of the NiO_6_ octahedra are always roughly twice the volume-shrink percentages of the NbO_6_ octahedra. Since the ratio of the NiO_6_ and NbO_6_ octahedra is 1:2 in the NiNb_2_O_6_ lattice, the expansion of the NiO_6_ octahedra almost completely offsets the shrinkage of the NbO_6_ octahedra through the reversible O movement, which is the “zero-strain” mechanism of NiNb_2_O_6_ in the broad temperature range. Undoubtedly, the electrochemical inactive NiO_6_ layers with superior volume-accommodation capability play the key role in achieving “zero-strain”.

## Conclusions

NiNb_2_O_6_ fibers constructed by nanosized primary particles (50–100 nm) are explored as an all-climate anode material with comprehensively good Li^+^-storage properties. This new NiNb_2_O_6_ material exhibits a high electronic conductivity (2.2 × 10^−8^ S cm^−1^), large Li^+^ diffusion coefficient (averaging at 1.1 × 10^−12^//6.1 × 10^−13^//1.3 × 10^−12^ cm^2^ s^−1^ at 25// − 10//60 °C), and significant pseudocapacitive behavior (94%//84%//82% capacitive contribution at 1.1 mV s^−1^ and 25// − 10//60 °C), leading to a large reversible capacity (300//184//318 mAh g^−1^ at 0.1C and 25// − 10//60 °C) and outstanding rate performance (10 to 0.5C capacity percentage of 64.3%//50.0%//65.4% at 25// − 10//60 °C) in a broad temperature range. The inactive NiO_6_ layers, which surround the active NbO_6_ layers, effectively accommodate the NbO_6_-volume change. The almost completely opposite volume changes of the NiO_6_ and NbO_6_ octahedra are achieved through the reversible O movement, leading to the “zero-strain” behavior of NiNb_2_O_6_ with minor unit-cell-volume change (0.53%//0.51%//0.74% at 25// − 10//60 °C) and excellent cyclability (90.1%//99.2%//92.3% capacity retention after 1000//2000//1000 cycles at 10C and 25// − 10//60 °C). The insight gained in this work can provide guide for the exploration of high-performance energy-storage materials working at harsh temperatures.

## Supplementary Information

Below is the link to the electronic supplementary material.

Supplementary file1 (DOCX 4868 kb)

Supplementary file2 (MP4 11601 kb)
