# Supplementary material for: “Zero-Strain” NiNb2O6 Fibers for All-Climate Lithium Storage
Source: Nanomicro Lett. 2024 Sep 27;17:15. doi: 10.1007/s40820-024-01497-z (PMC11427633; doi:10.1007/s40820-024-01497-z)
Supplement: Supplementary file 1 — Supplementary file1 (DOCX 4868 kb) [file 40820_2024_1497_MOESM1_ESM.docx]

Supporting Information for

**“Zero-Strain” NiNb_2_O_6_ Fibers for All-Climate Lithium Storage**

Yan Zhao^1, 2^, Qiang Yuan^2^, Liting Yang^3^, Guisheng Liang^3^, Yifeng Cheng^4^, Limin Wu^5,^ *, Chunfu Lin^1, 2,^ *, and Renchao Che^1, 3, 4,^ *

^1^ College of Physics, Donghua University, Shanghai 201620, P. R. China

^2^ Institute of Materials for Energy and Environment, School of Materials Science and Engineering, Qingdao University, Qingdao 266071, P. R. China

^3^ Laboratory of Advanced Materials, Shanghai Key Lab of Molecular Catalysis and Innovative Materials, Academy for Engineering & Technology, Fudan University, Shanghai 200438, P. R. China

^4^ Zhejiang Laboratory, Hangzhou 311100, P. R. China

^5^ Inner Mongolia University, Hohhot 010021, P. R. China

*Corresponding authors. E-mail addresses: [wlm@imu.edu.cn](mailto:wlm@imu.edu.cn) (Limin Wu); [linchunfu@qdu.edu.cn](mailto:linchunfu@qdu.edu.cn) (Chunfu Lin); [rcche@fudan.edu.cn](mailto:rcche@fudan.edu.cn) (Renchao Che)

**Supplementary Figures and Tables**


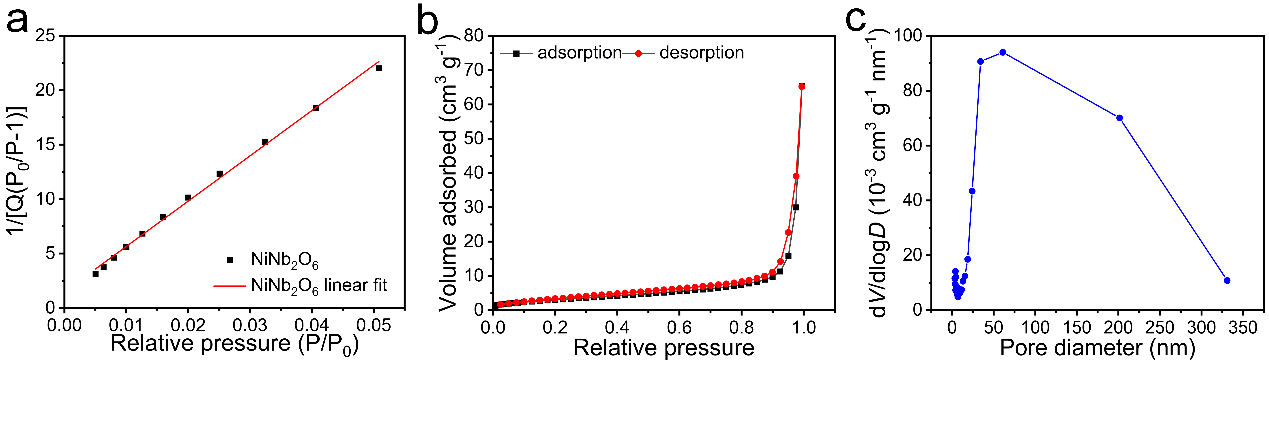


**Fig. S1** Physical adsorption test of NiNb_2_O_6_ fibers: **a**) BET specific surface area plot. **b**) N_2_ adsorption−desorption isotherm. **c**) BJH pore-size distribution curve from desorption branch


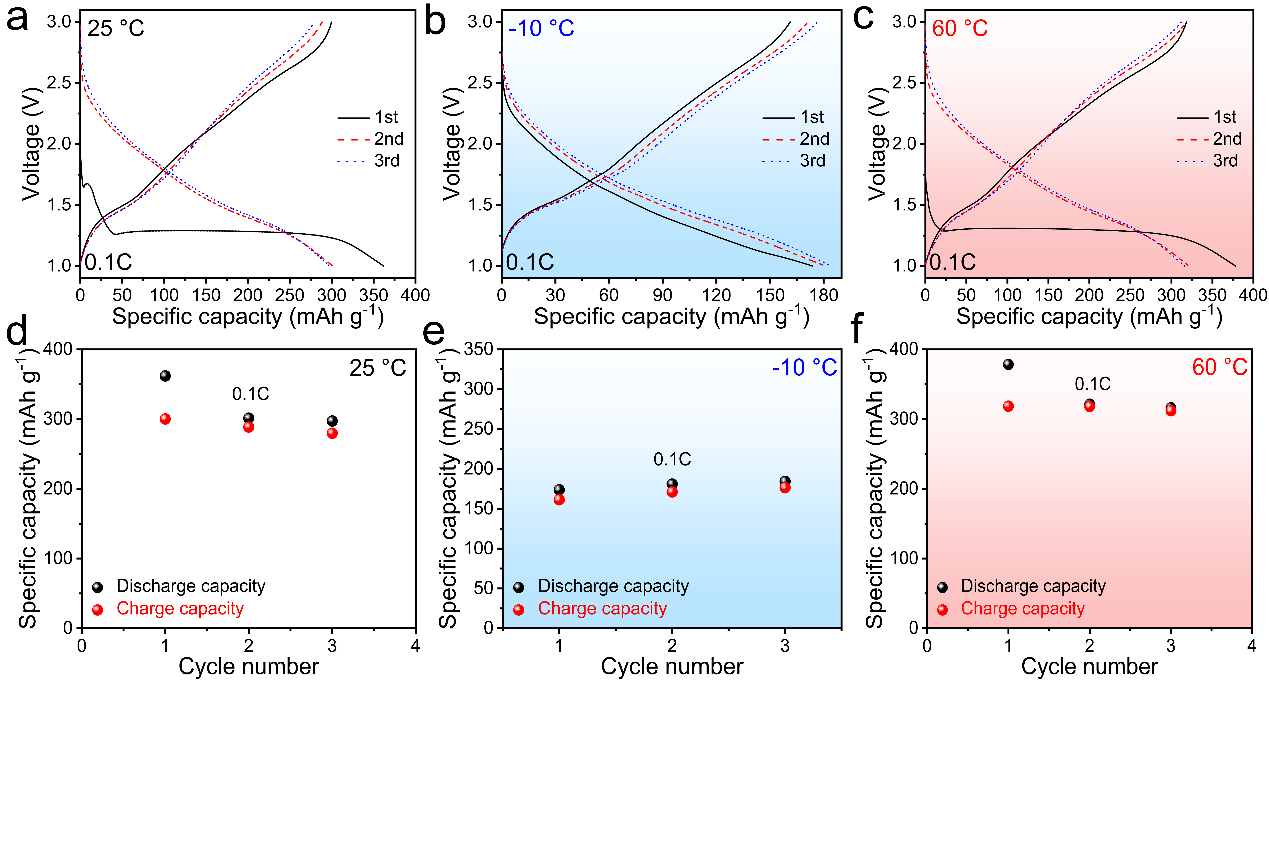


**Fig. S2** Electrochemical properties of NiNb_2_O_6_ at 0.1C and different temperatures. First three-cycle GCD profiles of NiNb_2_O_6_/Li half cells: **a**) 25, **b**) −10, and **c**) 60 °C. Reversible capacities of NiNb_2_O_6_/Li half cells: **d**) 25, **e**) −10, and **f**) 60 °C


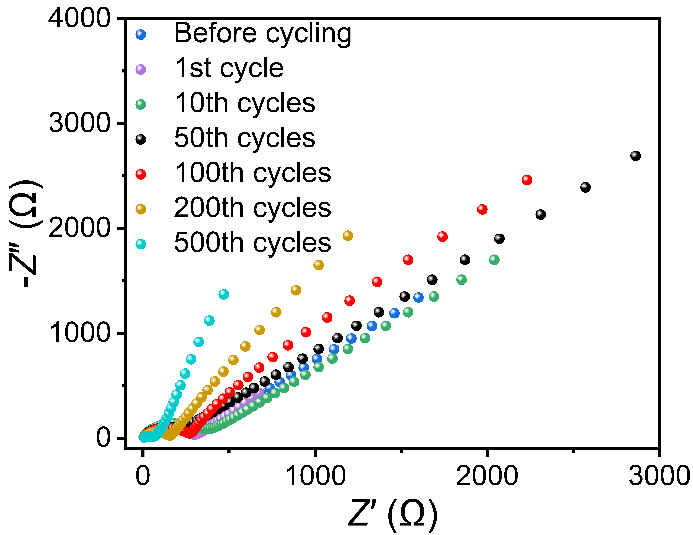


**Fig. S3** Nyquist plots of NiNb_2_O_6_/Li half cell after 0, 1, 10, 50, 100, 200, and 500 cycles at 25 °C


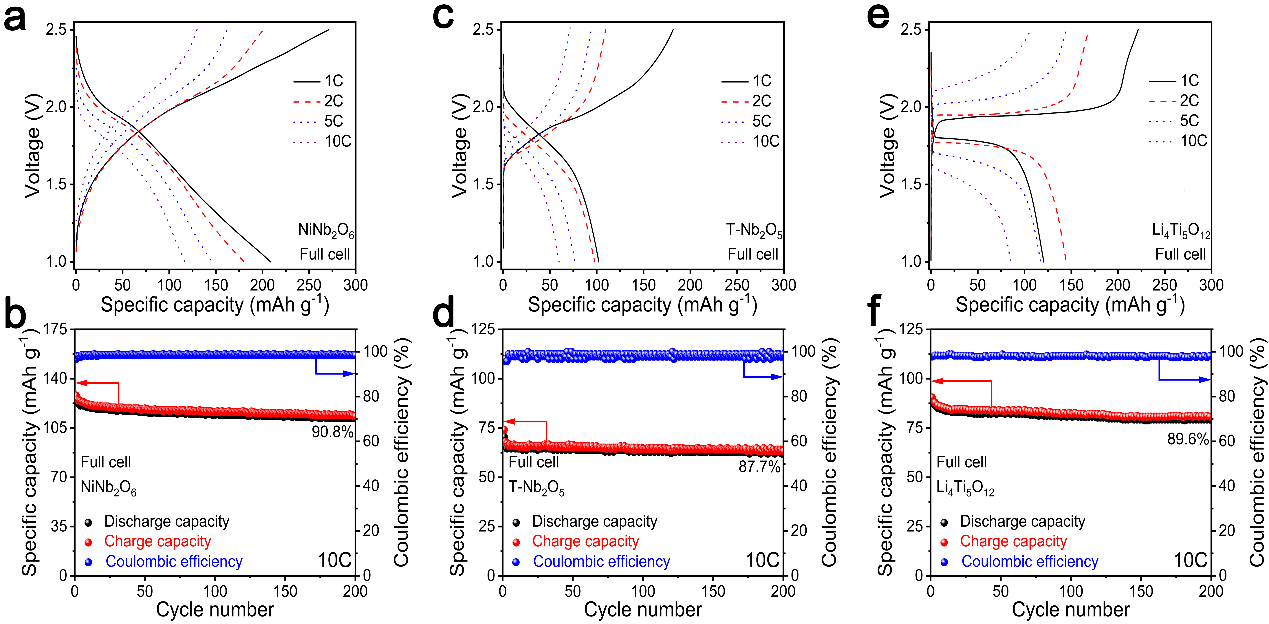


**Fig. S4** Electrochemical properties of full cells. **a**) GCD profiles and **b**) cyclability of LiFePO_4_/NiNb_2_O_6_ full cell. **c**) GCD profiles and **d**) cyclability of LiFePO_4_/T-Nb_2_O_5_ full cell. **e**) GCD profiles and **f**) cyclability of LiFePO_4_/Li_4_Ti_5_O_12_ full cell

The T-Nb_2_O_5_ nanoparticles were synthesized through a hydrothermal method. 1.1 g of C_10_H_5_NbO_20_ (Macklin, 98%) was dissolved in 30 mL of deionized water. After stirring at 50 °C for 1 h, the solution was transferred to a 50 mL autoclave, which was then exposed to a 150 °C oven for 12 h for a hydrothermal reaction. The white product after the reaction was collected and washed with deionized water. The obtained paste was vacuum-dried at 80 °C for 10 h and calcined at 700 °C for 4 h in air.

Active materials (NiNb_2_O_6_ fibers, T-Nb_2_O_5_, and commercial Li_4_Ti_5_O_12_ (LTO-1, Shenzhen BTR)), conductive agent (Super-P carbon) or binder (polyvinylidene fluoride) in a mass ratio of 7.5:1.5:1 was mixed in 1-methyl-2-pyrrolidinone. The formed slurries were cast on Cu foils. These electrode films were placed in a vacuum oven and dried at 110 °C for 10 h, forming the working electrodes with loadings of ≈1.0 mg cm^−2^. Full cells were assembled into CR2032-type coin cells. LiFePO_4_ (P198, Shenzhen BTR) and active materials with an N/P ratio of 0.95 (cathode-limited configuration) were used as cathode and anode materials, respectively. The fabrication of the LiFePO_4_ cathodes was similar to that of the above anodes, except that LiFePO_4_, Super-P carbon, and polyvinylidene fluoride in a mass ratio of 8:1:1 was coated on Al foils.

Compared to the LiFePO_4_/T-Nb_2_O_5_ and LiFePO_4_/Li_4_Ti_5_O_12_ full cells, the LiFePO_4_/NiNb_2_O_6_ full cell exhibits better electrochemical properties. Especially, the reversible capacity of the LiFePO_4_/NiNb_2_O_6_ full cell (212 mAh g^−1^ at 1C) is 100 and 60 mAh g^−1^ larger than that of the LiFePO_4_/T-Nb_2_O_5_ and LiFePO_4_/Li_4_Ti_5_O_12_ full cells, respectively.


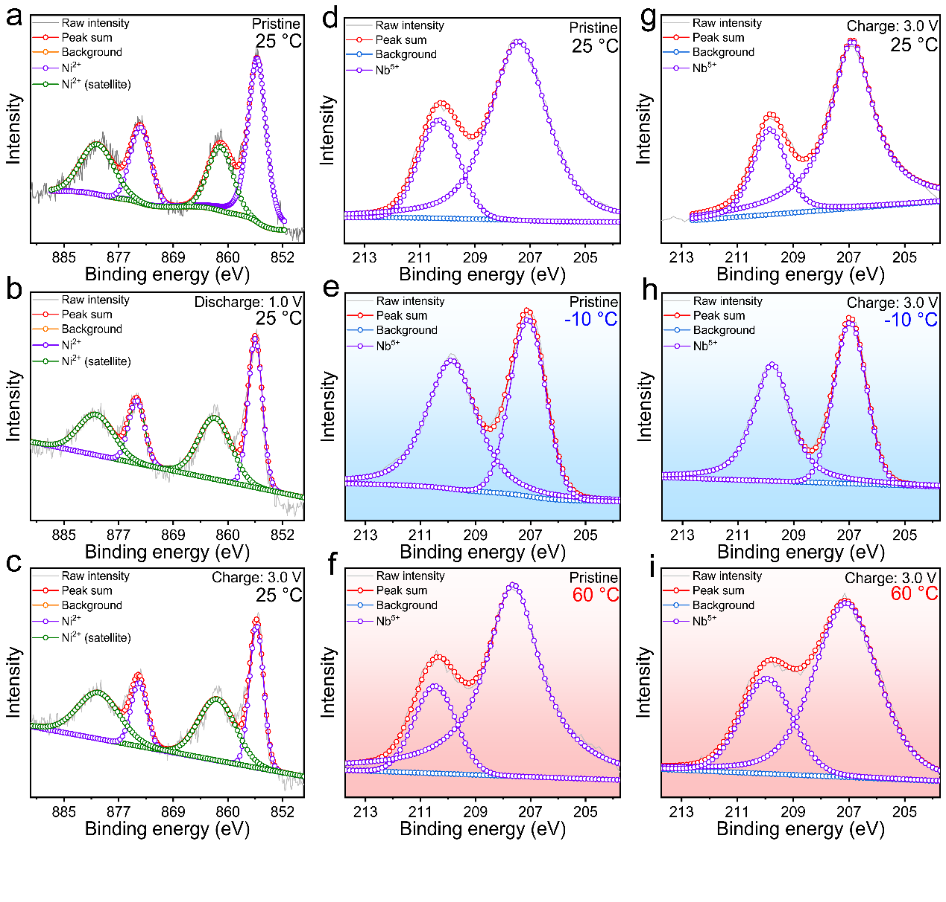


**Fig. S5** Redox mechanism of NiNb_2_O_6_ fibers. *Ex-situ* Ni-2*p* XPS spectra: **a**) pristine, **b**) discharge (1.0 V), and **c**) charge (3.0 V) states. *Ex-situ* Nb-3*d* XPS spectra at pristine state: **d**) 25, **e**) −10, and **f**) 60 °C. *Ex-situ* Nb-3*d* XPS spectra at charge (3.0 V) states: **g**) 25, **h**) −10, and **i**) 60 °C


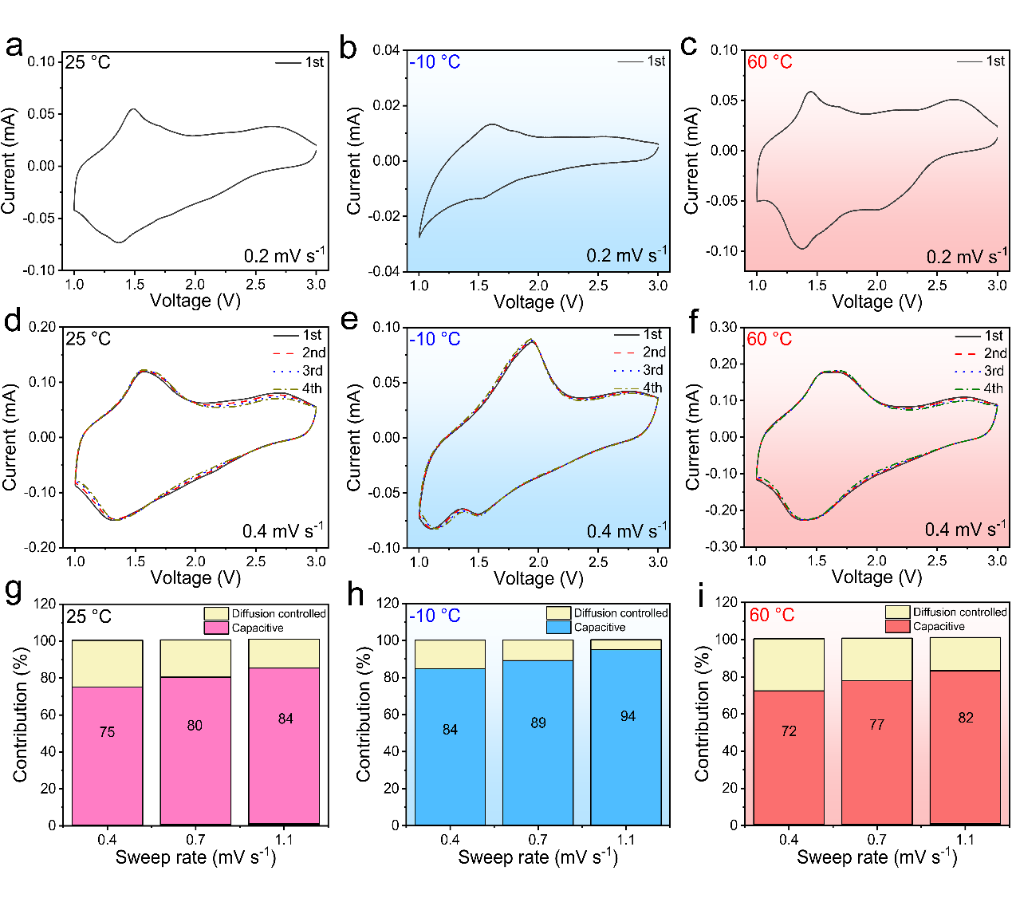


**Fig. S6** Electrochemical kinetics of NiNb_2_O_6_ fibers. CV profiles of NiNb_2_O_6_/Li half cell at 0.2 mV s^−1^: **a**) 25, **b**) −10, and **c**) 60 °C. CV profiles of NiNb_2_O_6_/Li half cell at 0.4 mV s^−1^: **d**) 25, **e**) −10, and **f**) 60 °C. Capacitive contributions of NiNb_2_O_6_/Li half cell at different sweep rates: **g**) 25, **h**) −10, and **i**) 60 °C


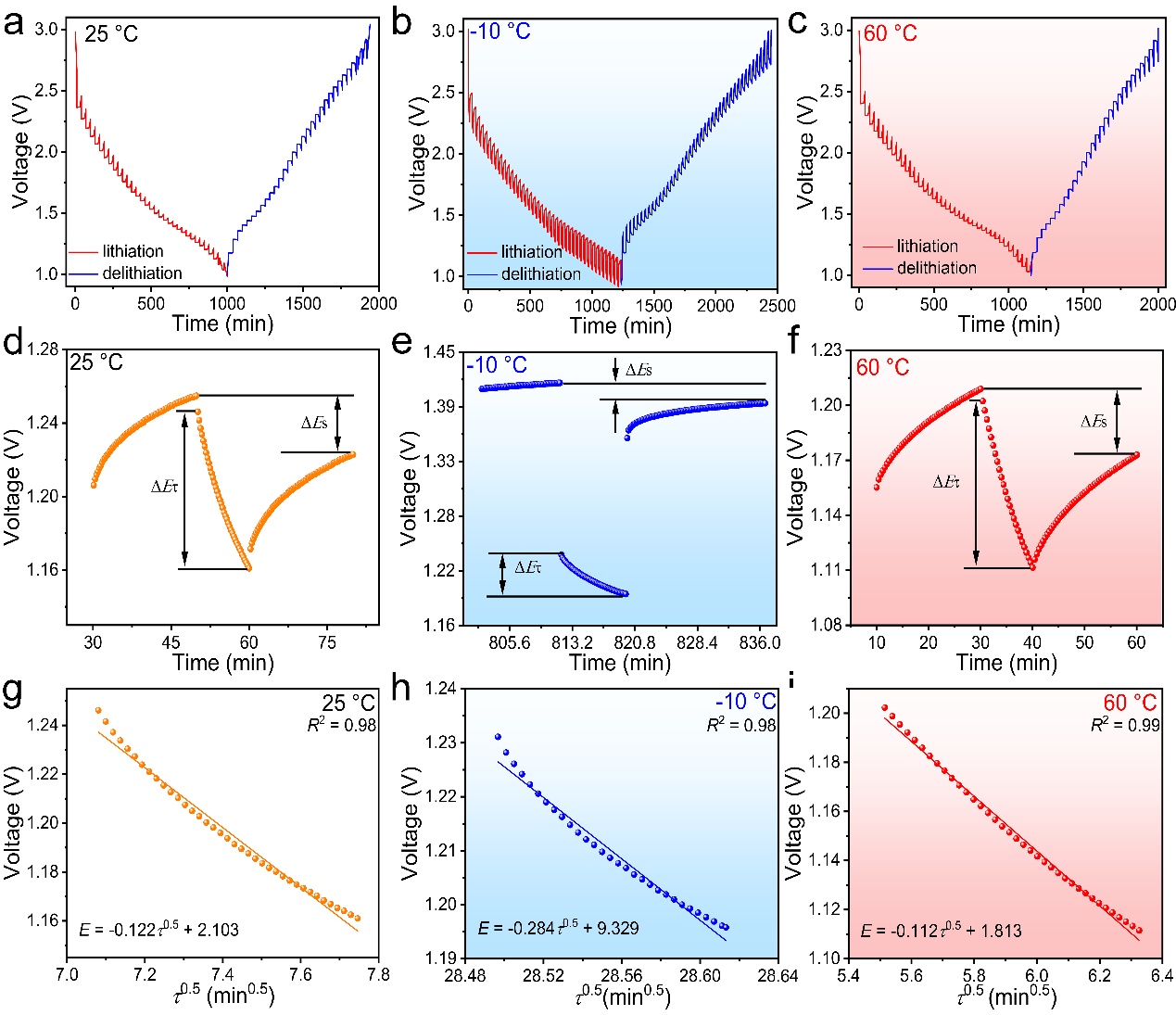


**Fig. S7** GITT tests of NiNb_2_O_6_ fibers. GITT curves (second cycle): **a**) 25, **b**) −10, and **c**) 60 °C. *E* versus *t* curves for a single step in GITT experiment: **d**) 25, **e**) −10, and **f**) 60 °C. Linear behavior of *E* versus *τ*^0.5^ relationship during a typical titration: **g**) 25, **h**) −10, and **i**) 60 °C

**Calculations of apparent Li^+^ diffusion coefficients of NiNb_2_O_6_ fibers from GITT**

The GITT tests are conducted on the NiNb_2_O_6_/Li cell to study the Li^+^ diffusivity in the NiNb_2_O_6_ fibers. Fig. S5a, Fig. S5b, and Fig. S5c respectively exhibit the typical GITT curves at 25, −10, and 60 °C during the first lithiation–delithiation cycle. For a clear observation, a single step of GITT is presented in Fig. S5d/Fig. S5e/Fig. S5f. Based on the Fick’s second law, the apparent Li^+^ diffusion coefficients (*D*_Li_) of the NiNb_2_O_6_ fibers can be calculated by using Eq. S1 [S1]:

$\text{D}_{\text{Li}}^{\text{ GITT}}\text{ =}\frac{\text{4}}{\text{π}}\left( \frac{\text{m}_{\text{b}}\text{V}_{\text{m}}}{\text{M}_{\text{b}}\text{S}} \right)^{\text{2}}\left( \frac{{\text{Δ}\text{E}}_{\text{s}}}{\text{τ}\left( {\text{d}\text{E}_{\text{τ}}}/{\text{d}\sqrt{\text{τ}}} \right)} \right)^{\text{2}}\text{ }\left( \text{τ}\text{≪}\frac{\text{L}^{\text{2}}}{\text{D}_{\text{Li}}} \right)$  (S1)

where, *M*_B_ is the molar mass of NiNb_2_O_6_, *V*_m_ is the molar volume of NiNb_2_O_6_, *m*_B_ is the mass of NiNb_2_O_6_, *S* is the NiNb_2_O_6_ electrode area, *τ* is the pulse duration time, *L* is the NiNb_2_O_6_ electrode thickness, and Δ*E*_s_ and Δ*E*_τ_ respectively represent the change in the equilibrium potential and the change in potential during the current pulse, which can be gained from the GITT curves (Fig. S5d/Fig. S5e/Fig. S5f). As the potential during a single titration delivers a linear relationship with *τ*^0.5^ (Fig. S5g/Fig. S5h/Fig. S5i), Eq. S1 can be simplified as Eq. S2:

$\text{D}_{\text{Li}}^{\text{ GITT}}\text{=}\frac{\text{4}}{\text{π}\text{τ}}\left( \frac{\text{m}_{\text{b}}\text{V}_{\text{m}}}{\text{M}_{\text{b}}\text{S}} \right)^{\text{2}}\left( \frac{{\text{Δ}\text{E}}_{\text{s}}}{{\text{Δ}\text{E}}_{\text{τ}}} \right)^{\text{2}} \left( \text{τ}\text{≪}\frac{\text{L}^{\text{2}}}{\text{D}_{\text{Li}}} \right)$ (S2)

Based on Eq. S2, the apparent Li^+^ diffusion coefficients of the NiNb_2_O_6_ fibers during different states of discharge/charge at 25, −10, and 60 °C are obtained, and displayed in Fig. 3g-i, respectively.


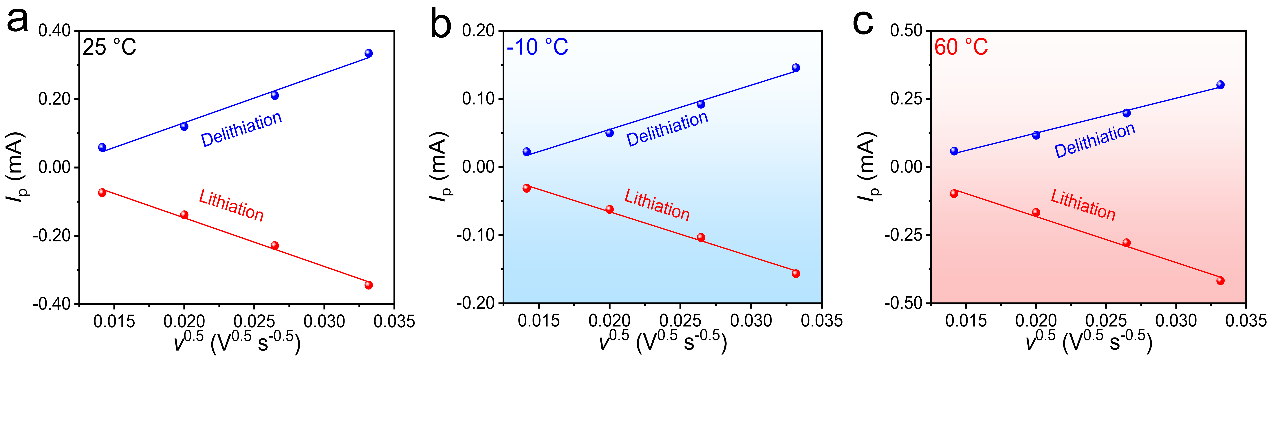


**Fig. S8** Relationship between peak current (*I*_p_) and square root of sweep rate (*v*^0.5^) for intensive cathodic and anodic peaks of NiNb_2_O_6_/Li half cells: **a**) 25, **b**) −10, and **c**) 60 °C

**Calculations of apparent Li^+^ diffusion coefficients of** **NiNb_2_O_6_ fibers by CV**

The apparent Li^+^ diffusion coefficients of the NiNb_2_O_6_ fibers at 25, −10, and 60 °C are also determined from the CV data at different sweep rates (Fig. 3d, Fig. 3e, and Fig. 3f). It is found that the peak current of the intensive cathodic/anodic reaction *I*_p_ is in proportional to the square root of the sweep rate *v*^0.5^ (Fig. S6), which shows the linear semi-infinite diffusion in cathodic and anodic processes. Consequently, the Randles−Sevcik equation (Eq. S3) can be applied, based on which the Li^+^ diffusion coefficient *D*_Li_ can be calculated [S2].

*I*_p_ = 2.69×10^5^*Sn*^1.5^*C*${\text{(}\text{D}}_{\text{Li}}^{\text{ }\text{CV}}$*v*)^0.5^ (S3)

where, *S* is the electrode area, *n* is the charge transfer number, and *C* is the molar concentration of Li^+^ in solid. The obtained *D*_Li_ values of NiNb_2_O_6_ fibers reach 2.3×10^−13^ cm^2^ s^−1^ (lithiation) and 3.0×10^−13^ cm^2^ s^−1^ (delithiation) at 25 °C, decrease to 1.0×10^−13^ cm^2^ s^−1^ (lithiation) and 1.1×10^−13^ cm^2^ s^−1^ (delithiation) at −10 °C, and increase to 4.2×10^−13^ cm^2^ s^−1^ (lithiation) and 7.3×10^−13^ cm^2^ s^−1^ (delithiation) at 60 °C, which match with the GITT results.


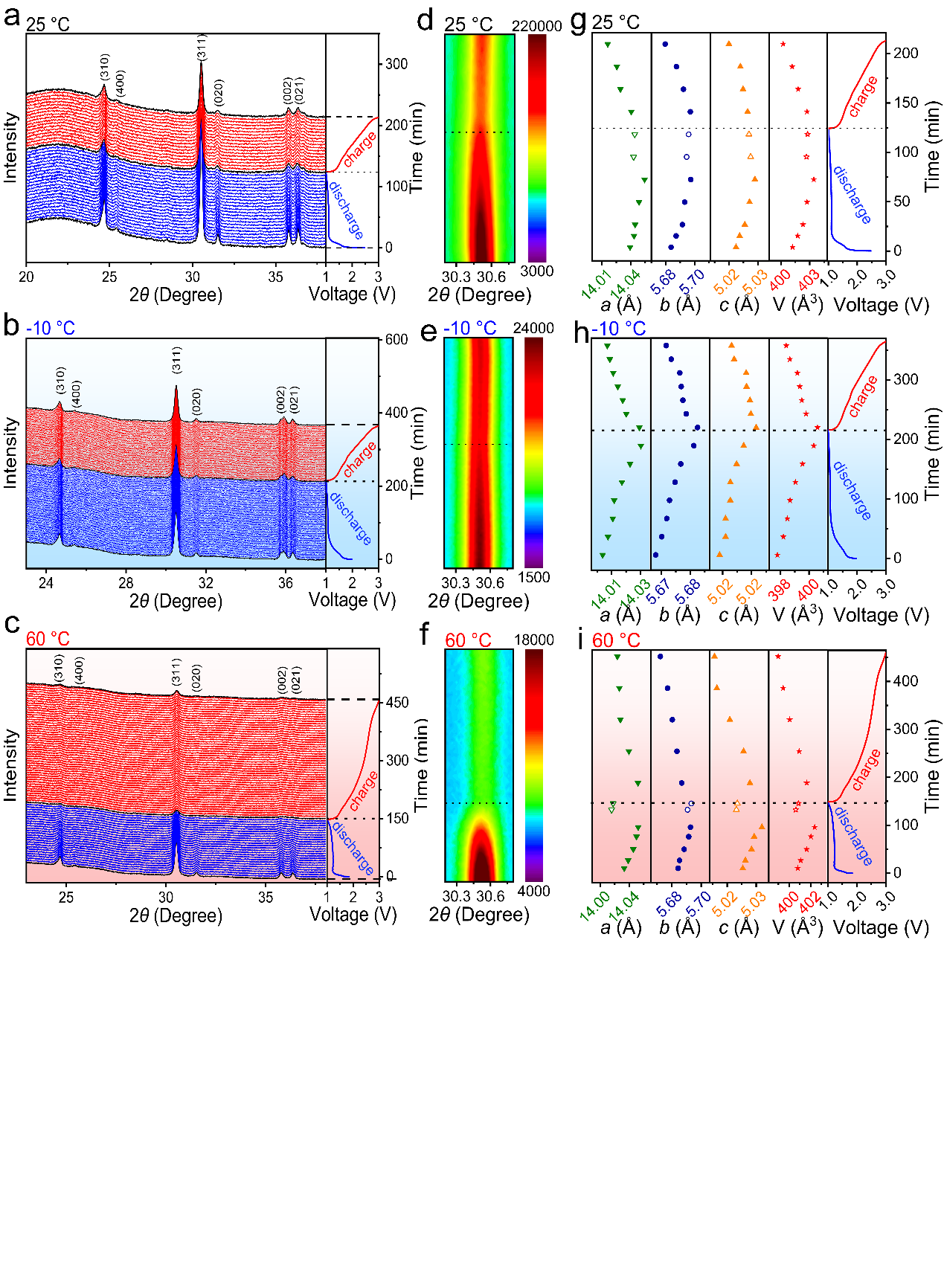


**Fig. S9** *In-situ* XRD characterizations of NiNb_2_O_6_ fibers within 1.0−3.0 V (first cycle at 0.5C and 25 °C, at 0.1C and −10 °C, and at 0.5C and 60 °C). *In-situ* XRD patterns of NiNb_2_O_6_/Li half cell with discharge−charge curves: **a**) 25, **b**) −10, and **c**) 60 °C. 2D *in-situ* XRD patterns enlarged within 30.3−30.7°: **d**) 25, **e**) −10, and **f**) 60 °C. Variations in lattice parameters of NiNb_2_O_6_ during discharge−charge: **g**) 25, **h**) −10, and **i**) 60 °C. Solid and hollow symbols represent data for original phase and transformed phase, respectively


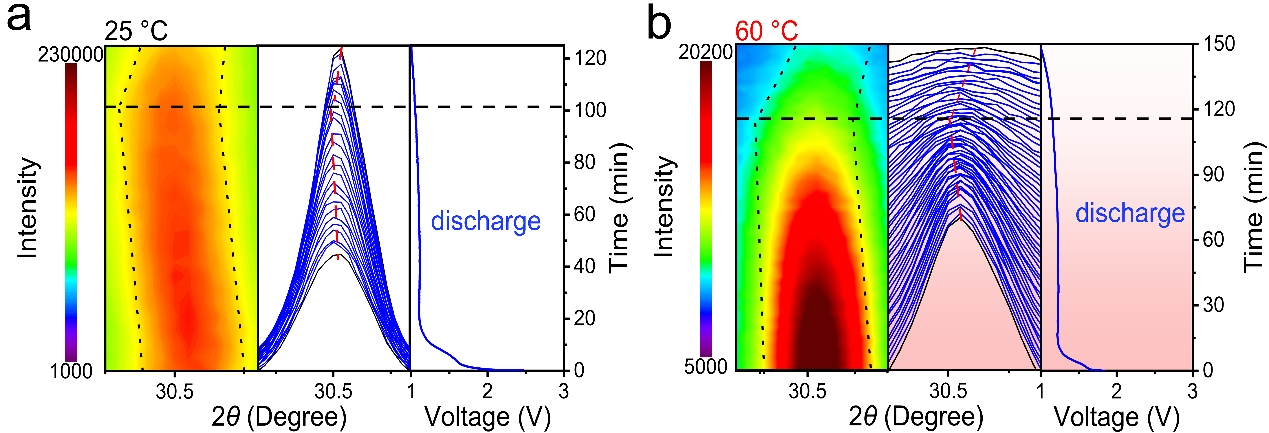


**Fig. S10** Sudden movement of (311) peak after phase transformation from *in-situ* XRD patterns of NiNb_2_O_6_/Li half cell (first discharge): **a**) 25 and **b**) 60 °C

**
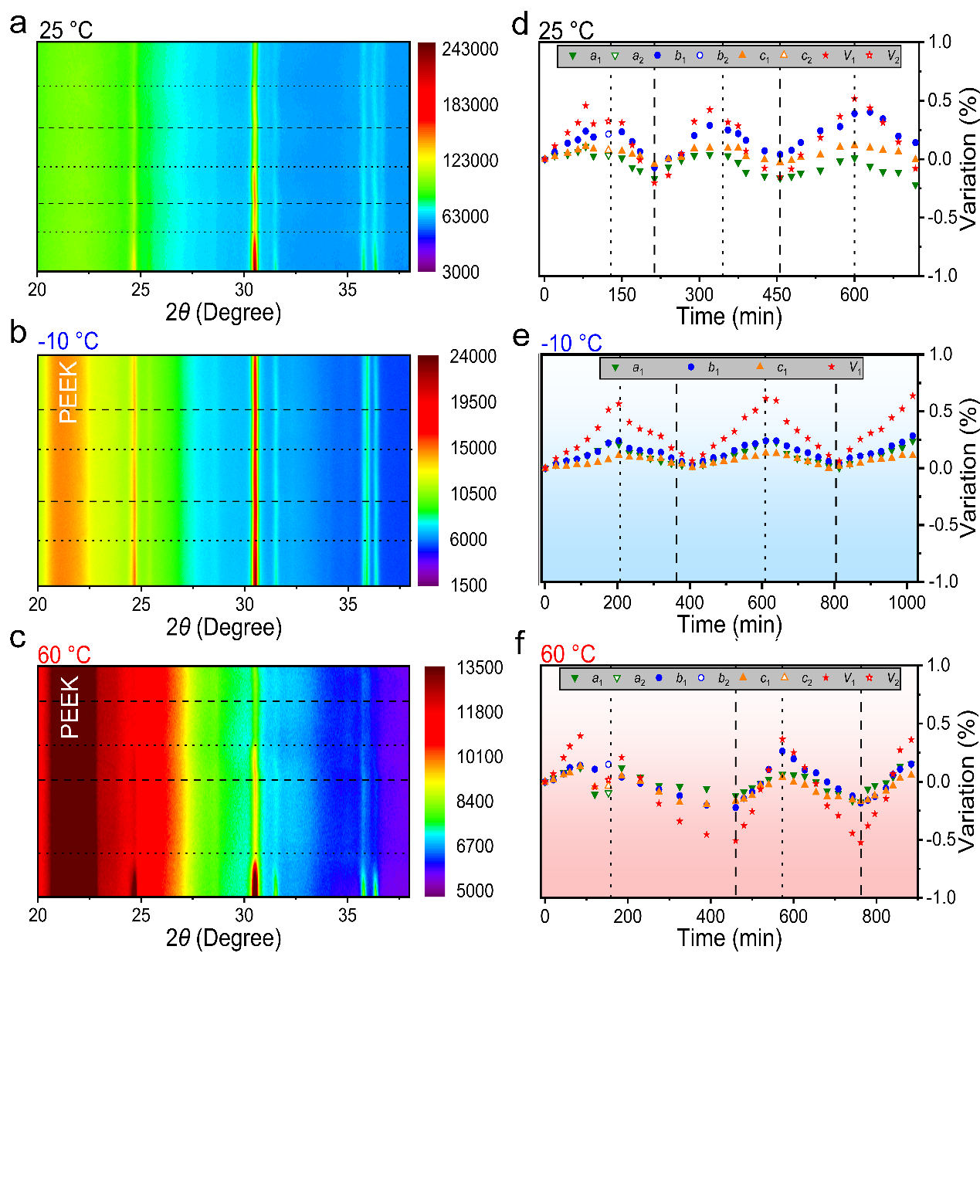
**

**Fig. S11** *In-situ* XRD characterizations of NiNb_2_O_6_ fibers within 1.0−3.0 V (first four cycles at 0.5C and 25 °C, at 0.1C and −10 °C, and at 0.5C and 60 °C). 2D *in-situ* XRD patterns of NiNb_2_O_6_/Li half cell: **a**) 25, **b**) −10, and **c**) 60 °C. Variations in lattice constants of NiNb_2_O_6_: **d**) 25, **e**) −10, and **f**) 60 °C. PEEK: polyetheretherketone. Solid and hollow symbols represent data for original phase and transformed phase, respectively


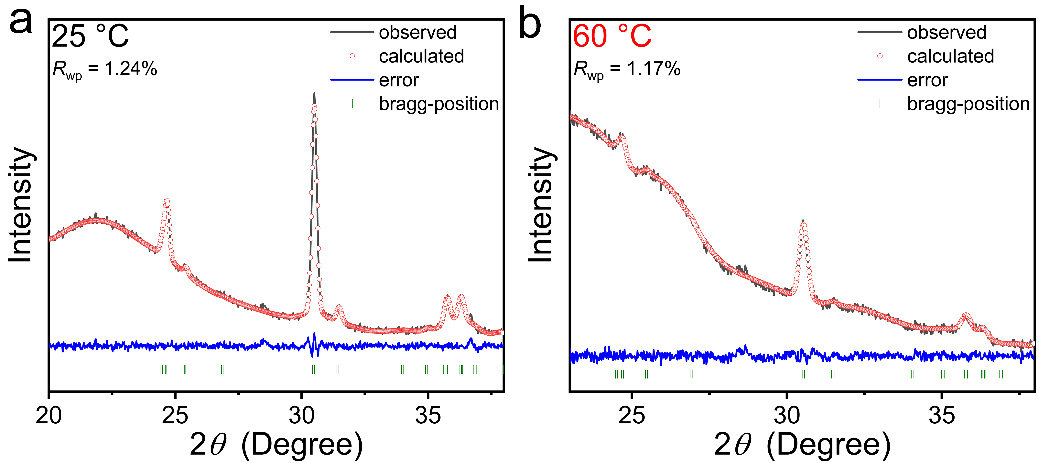


**Fig. S12** Rietveld-refined *operando* XRD patterns of NiNb_2_O_6_/Li operando cell at 1.0 V: **a**) 25 and **b**) 60 °C


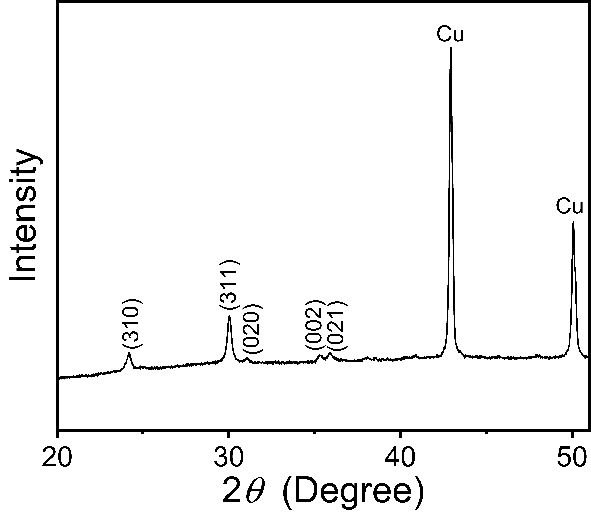


**Fig. S13** *Ex-situ* XRD pattern of NiNb_2_O_6_ electrode after 100 cycles


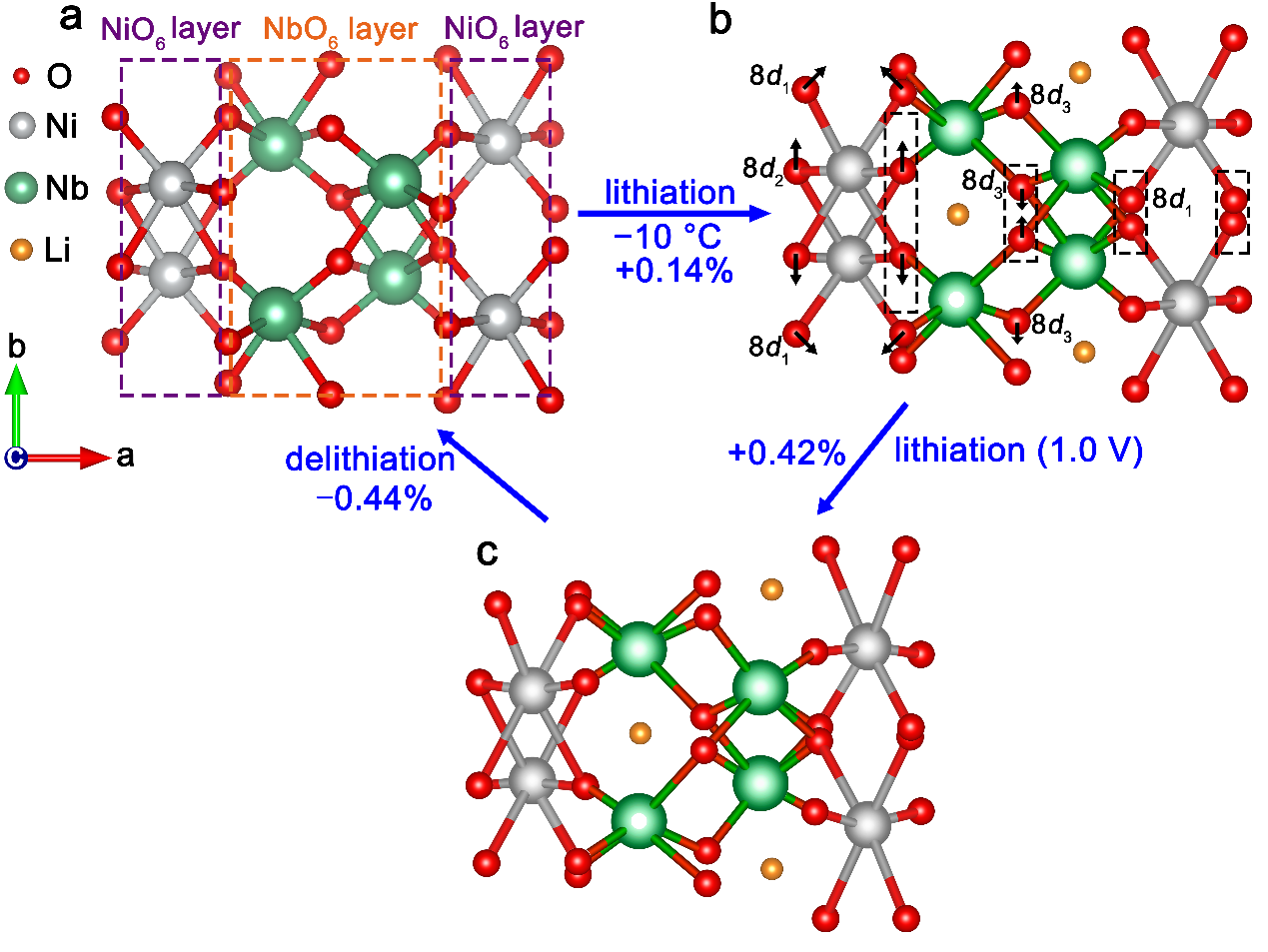


**Fig. S14** “Zero-strain” mechanism of NiNb_2_O_6_ at −10 °C. Movement of ions from (**a**) initial stage to (**b**) partially lithiated stage, and finally to (**c**) final lithiated stage. Li, Ni, Nb, and O are colored by orange, grey, green, and red, respectively. Arrow lengths indicate movement distances


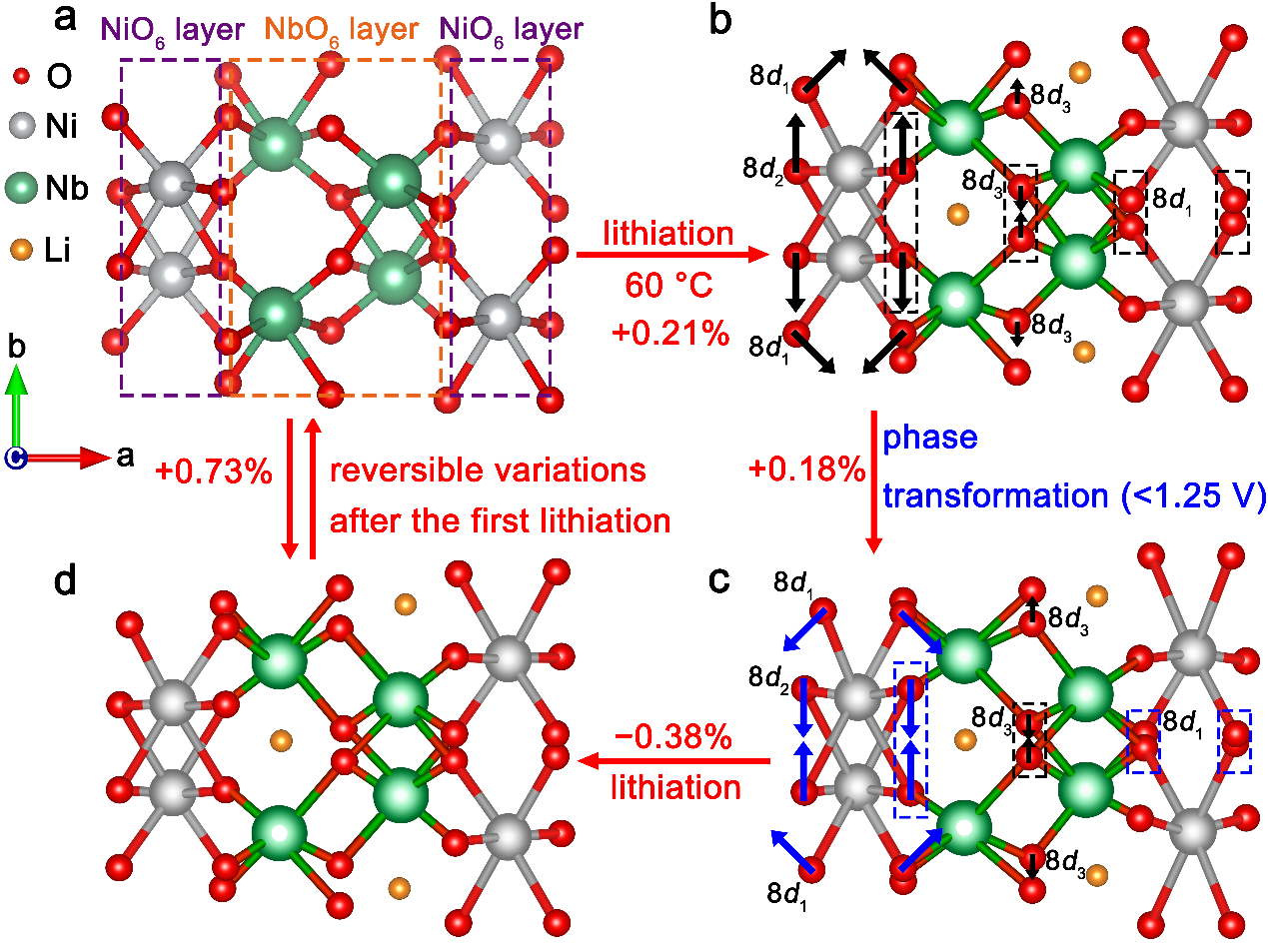


**Fig. S15** “Zero-strain” mechanism of NiNb_2_O_6_ at 60 °C. Movement of ions from (**a**) initial stage to (**b**) partially lithiated stage, then to (**c**) phase-transformed stage, and finally to (**d**) final lithiated stage. Li, Ni, Nb, and O are colored by orange, grey, green, and red, respectively. Arrow lengths indicate movement distances.

**Table S1** Comparisons of electrochemical properties of NiNb_2_O_6_ with previously-reported anode materials with intercalation characteristic

| Material | Rate capability | Cyclic stability | References |
| --- | --- | --- | --- |
| **NiNb_2_O_6_ fibers** | **164 mAh g^−1^ at 10C** | **capacity retention of 92.8% over 1000 cycles at 10C** | **this work** |
| NiNb_2_O_6_ micron-sized particles | 126 mAh g^−1^ at 10C | capacity retention of 92.0% over 2500 cycles at 10C | [S3] |
| partially reduced TiNb_24_O_62_ nanofibers | 190 mAh g^−1^ at 10C | capacity retention of 86.4% over 500 cycles at 10C | [S4] |
| GaNb_11_O_29_ micron-sized particles | 121 mAh g^−1^ at 10C | capacity retention of 87.5% over 1000 cycles at 10C | [S5] |
| reduced Mo*_x_*Ti_1−_*_x_*Nb_2_O_7+_*_y_* micron-sized particles | 192 mAh g^−1^ at 12C | capacity retention of 73.0% over 500 cycles at 12C | [S6] |
| 2D Nb_2_O_5_-C-rGO micron-sized sheets | ~165 mAh g^−1^ at 10C | capacity retention of 78.0% over 1500 cycles at 5C | [S7] |
| TiNb_2_O_7_ hierarchical microspheres | 100 mAh g^−1^ at 20C | capacity retention of 83.5% over 500 cycles at 10C | [S8] |
| Ti_2_Nb_10_O_29_ hollow nanofibers | 136 mAh g^−1^ at 20C | capacity retention of ~70% over 500 cycles at 10C | [S9] |
| TiNb_2_O_7_ nanoparticles | 87 mAh g^−1^ at 30C | capacity retention of ~83% over 500 cycles at 10C | [S10] |
| W_3_Nb_14_O_44_ nanowires | 131 mAh g^−1^ at 5C | capacity retention of ~64% over 1000 cycles at 10C | [S11] |
| Li_4_Ti_5_O_12_ nanorods | 136 mAh g^−1^ at 20C | capacity retention of ~61.5% over 1500 cycles at 10C | [S12] |
| Mo_1.5_W_1.5_Nb_14_O_44_ micron-sized particles | 198 mAh g^−1^ at 10C | capacity retention of 84.1% over 2000 cycles at 10C | [S13] |
| Nb_14_W_3_O_44_ nano-blocks | 160 mAh g^−1^ at 10C | capacity retention of 84.3% over 1000 cycles at 10C | [S14] |
| Cr_0.2_Fe_0.8_Nb_11_O_29_ micron-sized particles | 123 mAh g^−1^ at 10C | capacity retention of 86.9% over 500 cycles at 10C | [S15] |
| HfNb_24_O_62_ micron-sized particles | 78 mAh g^−1^ at 10C | capacity retention of 87.1% over 500 cycles at 10C | [S16] |
| 3D TiO_2_-QDs@C porous nanospheres | 108 mAh g^−1^ at 5A g^−1^ | capacity retention of 68.6% over 10000 cycles at 5A g^−1^ | [S17] |
| Mg_2_Nb_34_O_87_ micron-sized particles | 117 mAh g^−1^ at 10C | capacity retention of 83.9% over 2000 cycles at 10C | [S18] |
| AlNb_11_O_29_ micron-sized particles | 131 mAh g^−1^ at 10C | capacity retention of 93.2% over 500 cycles at 10C | [S19] |
| graphite micron-sized particles | 135 mAh g^−1^ at 10C | capacity retention of 73.0% over 1500 cycles at 10C | [S20] |
| carbon-coated Li_3_VO_4_ | 147 mAh g^−1^ at 0.3 A g^−1^ | 84.5% after 50 cycles at 0.02 A g^−1^ | [S21] |
| *ω*-Li_3_V_2_O_5_ nanoplates | 113 mAh g^−1^ at 10 A g^−1^ | 99.5% after 1000 cycles at 10 A g^−1^ | [S22] |
| Li_2.98_Cr_0.04_V_0.98_O_4_ microparticles | 147 mAh g^−1^ at 1.2 A g^−1^ | 86.6% after 1000 cycles at 1.2 A g^−1^ | [S23] |

**Table S2** Comparisons of apparent Li^+^ diffusion coefficient (*D*_Li_) of NiNb_2_O_6_ with previously-reported M−Nb−O anode materials

| Material | *D*_Li_ (cm^2^ s^−1^) | Test technique | References |
| --- | --- | --- | --- |
| **NiNb_2_O_6_ fibers (25 °C)** | **1.11×10^−12^** | **GITT** | **this work** |
| **NiNb_2_O_6_ fibers (−10 °C)** | **6.12×10^−13^** | **GITT** | **this work** |
| **NiNb_2_O_6_ fibers (60 °C)** | **1.29×10^−13^** | **GITT** | **this work** |
| **NiNb_2_O_6_ fibers (25 °C)** | **2.67×10^−13^** | **CV** | **this work** |
| **NiNb_2_O_6_ fibers (−10 °C)** | **1.10×10^−13^** | **CV** | **this work** |
| **NiNb_2_O_6_ fibers (60 °C)** | **5.73×10^−13^** | **CV** | **this work** |
| NiNb_2_O_6_ micron-sized particles (25 °C) | 1.36×10^−12^ | GITT | [S3] |
| W_3_Nb_14_O_44_ nanowires (25 °C) | 8.02×10^−13^ | CV | [S11] |
| Cu_2_Nb_34_O_87_ micron-sized particles (25 °C) | 3.50×10^−13^ | GITT | [S24] |
| VNb_9_O_25_ nanoribbons (25 °C) | 5.17×10^−15^ | EIS | [S25] |
| MoNb_12_O_33_ micron-sized particles (25 °C) | 3.90×10^−14^ | GITT | [S26] |
| Al_0.5_Nb_24.5_O_62_ micron-sized particles (25 °C) | 2.50×10^−13^ | GITT | [S27] |
| GeNb_18_O_47_ nanowires (25 °C) | 1.57×10^−14^ | CV | [S28] |
| W_5_Nb_16_O_55_ micron-sized particles (25 °C) | 1.0×10^−13^ | GITT | [S29] |
| TiNb_6_O_17_ micron-sized particles (25 °C) | 4.88×10^−14^ | CV | [S30] |
| TiCr_0.5_Nb_10.5_O_29_ nanoparticles | 2.07×10^−14^ | CV | [S31] |
| TiNb_2_O_7_ micron-sized particles (25 °C) | 1.05×10^−15^ | EIS | [S8] |
| HfNb_24_O_62_ micron-sized particles (25 °C) | 1.51×10^−13^ | GITT | [S16] |
| W_16_Nb_18_O_93_ nanowires (25 °C) | 1.31×10^−14^ | EIS | [S32] |
| TiO_2_/C composite nanospheres (25 °C) | 2.2×10^−14^ | CV | [S17] |
| Cr_0.5_Nb_24.5_O_62_ micron-sized particles (25 °C) | 4.57×10^−14^ | EIS | [S33] |
| TiNb_2_O_7_ nanorods (25 °C) | 3.24×10^−14^ | CV | [S34] |
| Nb_2_O_5_ nanorods (25 °C) | 3.66×10^−17^ | CV | [S35] |

**Table S3** Comparisons of maximum unit-cell-volume change of NiNb_2_O_6_ with previously-reported M−Nb−O anode materials

| Material | Maximum unit-cell-volume change (%) | References |
| --- | --- | --- |
| **NiNb_2_O_6_ (25 °C)** | **0.53 (discharge to 1.0 V)** | **this work** |
| **NiNb_2_O_6_ (−10 °C)** | **0.51 (discharge to 1.0 V)** | **this work** |
| **NiNb_2_O_6_ (60 °C)** | **0.74 (discharge to 1.0 V)** | **this work** |
| LaCeNb_6_O_18_ (25 °C) | 1.52 (discharge to 0.8 V) | [S36] |
| V_3_Nb_17_O_12_ (25 °C) | 3.46 (discharge to 0.8 V) | [S37] |
| BaNb_3.6_O_10_ (25 °C) | 5.96 (discharge to 0.8 V) | [S38] |
| NaNb_13_O_33_ (25 °C) | 6.02 (discharge to 0.8 V) | [S39] |
| Ni_2_Nb_34_O_87_ (25 °C) | 6.71 (discharge to 0.8 V) | [S40] |
| Ti_2_Nb_10_O_29_ (25 °C) | 6.8 (discharge to 1.0 V) | [S9] |
| GeNb_18_O_47_ (25 °C) | 6.83 (discharge to 0.8 V) | [S28] |
| TiNb_2_O_7_ (25 °C) | 7.22 (discharge to 1.0 V) | [S10] |
| MoNb_13_O_33_ (25 °C) | 7.8 (discharge to 0.8 V) | [S26] |
| Cu_2_Nb_34_O_87_ (25 °C) | 8.32 (discharge to 0.8 V) | [S24] |
| Al_0.5_Nb_24.5_O_62_ (25 °C) | 8.5 (discharge to 0.8 V) | [S27] |
| Zn_2_Nb_34_O_87_ (25 °C) | 8.58 (discharge to 0.8 V) | [S41] |
| VNb_9_O_25_ (25 °C) | 8.91 (discharge to 0.8 V) | [S25] |
| Mo_3_Nb_14_O_44_ (25 °C) | 10.6 (discharge to 0.8 V) | [S42] |
| PNb_9_O_25_ (25 °C) | 10.69 (discharge to 0.8 V) | [S43] |
| Mo_4_Nb_26_O_77_ (25 °C) | 12.8 (discharge to 0.8 V) | [S44] |

**Table S4** Lattice parameters of delithiated NiNb_2_O_6_, partially lithiated NiNb_2_O_6_, lithiated NiNb_2_O_6_ after phase transformation, and lithiated NiNb_2_O_6_ states at 25 °C

| sample | *a* (Å) | *b* (Å) | *c* (Å) | *V* (Å^3^) | space group | *R*_wp_ | Uiso |
| --- | --- | --- | --- | --- | --- | --- | --- |
| delithiated NiNb_2_O_6_ | 14.03928  (58) | 5.68370  (16) | 5.02556  (12) | 401.015  (37) | *Pbcn* | 0.0152 | 0.01 |
| partially lithiated NiNb_2_O_6_ | 14.04520  (56) | 5.69151  (32) | 5.02825  (47) | 401.940  (78) | *Pbcn* | 0.0132 | 0.01 |
| lithiated NiNb_2_O_6_ after phase transformation | 14.05438  (84) | 5.69734  (24) | 5.03114  (18) | 402.839  (54) | *Pbcn* | 0.0126 | 0.01 |
| lithiated NiNb_2_O_6_ | 14.04300  (11) | 5.69445  (32) | 5.02986  (24) | 402.223  (72) | *Pbcn* | 0.0124 | 0.01 |

**Table S5** Fractional atomic parameters of delithiated NiNb_2_O_6_ at 25 °C

| ion | site | *x* | *y* | *z* |
| --- | --- | --- | --- | --- |
| Ni^2+^ | 4*c* | 0 | 0.3324(14) | 0.25 |
| Nb^5+^ | 8*d* | 0.1604(62) | 0.1807(57) | 0.7482(28) |
| O^2−^ | 8*d*_1_ | 0.0875(40) | 0.0807(43) | 0.0910(42) |
| O^2−^ | 8*d*_2_ | 0.4205(11) | 0.1248(40) | 0.0867(35) |
| O^2−^ | 8*d*_3_ | 0.7569(12) | 0.1199(39) | 0.0770(43) |

**Table S6** Fractional atomic parameters of partially lithiated NiNb_2_O_6_ at 25 °C

| ion | site | *x* | *y* | *z* |
| --- | --- | --- | --- | --- |
| Ni^2+^ | 4*c* | 0 | 0.3328(18) | 0.25 |
| Nb^5+^ | 8*d* | 0.1594(79) | 0.1802(48) | 0.7598(42) |
| O^2−^ | 8*d*_1_ | 0.0847(21) | 0.0643(04) | 0.1205(23) |
| O^2−^ | 8*d*_2_ | 0.4211(14) | 0.1460(51) | 0.0796(43) |
| O^2−^ | 8*d*_3_ | 0.7558(27) | 0.1090(19) | 0.0820(52) |

**Table S7** Fractional atomic parameters of lithiated NiNb_2_O_6_ after phase transformation at 25 °C

| ion | site | *x* | *y* | *z* |
| --- | --- | --- | --- | --- |
| Ni^2+^ | 4*c* | 0 | 0.3304(16) | 0.25 |
| Nb^5+^ | 8*d* | 0.1606(18) | 0.1815(62) | 0.7654(09) |
| O^2−^ | 8*d*_1_ | 0.0875(76) | 0.0599(54) | 0.1129(10) |
| O^2−^ | 8*d*_2_ | 0.4211(56) | 0.1399(26) | 0.0867(35) |
| O^2−^ | 8*d*_3_ | 0.7546(08) | 0.1045(62) | 0.0788(17) |

**Table S8** Fractional atomic parameters of lithiated NiNb_2_O_6_ at 25 °C

| ion | site | *x* | *y* | *z* |
| --- | --- | --- | --- | --- |
| Ni^2+^ | 4*c* | 0 | 0.3269(22) | 0.25 |
| Nb^5+^ | 8*d* | 0.1608(10) | 0.1825(92) | 0.7673(29) |
| O^2−^ | 8*d*_1_ | 0.0900(16) | 0.0552(74) | 0.1138(70) |
| O^2−^ | 8*d*_2_ | 0.4215(16) | 0.1297(66) | 0.0965(55) |
| O^2−^ | 8*d*_3_ | 0.7531(18) | 0.0995(72) | 0.0749(77) |

**Table S9** Bond lengths of delithiated NiNb_2_O_6_, partially lithiated NiNb_2_O_6_, lithiated NiNb_2_O_6_ after phase transformation, and lithiated NiNb_2_O_6_ states at 25 °C

| bond | delithiated NiNb_2_O_6_ (Å) | partially lithiated NiNb_2_O_6_ (Å) | lithiated NiNb_2_O_6_ after phase transformation (Å) | lithiated NiNb_2_O_6_ (Å) |
| --- | --- | --- | --- | --- |
| Ni−O (8*d*_1_) | 2.0890(5) | 2.0894(6) | 2.1120(7) | 2.0820(8) |
| Ni−O (8*d*_2_) | 2.1290(5) | 2.1146(6) | 2.1020(7) | 2.1357(8) |
| Nb−O (8*d*_1_) | 2.0109(5) | 2.0267(6) | 2.0374(6) | 1.9910(7) |
| Nb−O (8*d*_2_) | 1.7811(5) | 1.7777(6) | 1.7546(6) | 1.7942(6) |
| Nb−O (8*d*_3_) | 2.0833(5) | 2.0907(6) | 2.0915(6) | 2.0929(7) |

**Table S10** NiO_6_ and NbO_6_ polyhedron-volumes in delithiated NiNb_2_O_6_, partially lithiated NiNb_2_O_6_, lithiated NiNb_2_O_6_ after phase transformation, and lithiated NiNb_2_O_6_ states at 25 °C

| sample | NiO_6_ (Å^3^) | NbO_6_ (Å^3^) |
| --- | --- | --- |
| delithiated NiNb_2_O_6_ | 11.9050 | 10.4227 |
| partially lithiated NiNb_2_O_6_ | 12.0946 | 10.3599 |
| lithiated NiNb_2_O_6_ after the phase transformation | 12.4262 | 10.2432 |
| lithiated NiNb_2_O_6_ | 12.4922 | 10.1900 |

**Table S11** Lattice parameters of delithiated and lithiated NiNb_2_O_6_ at −10 °C

| sample | *a* (Å) | *b* (Å) | *c* (Å) | *V* (Å^3^) | Space group | *R_wp_* | Uiso |
| --- | --- | --- | --- | --- | --- | --- | --- |
| delithiated NiNb_2_O_6_ | 13.99453  (11) | 5.66938  (37) | 5.01653  (21) | 398.013  (75) | *Pbcn* | 0.0153 | 0.01 |
| lithiated NiNb_2_O_6_ | 14.02392  (11) | 5.68242(37) | 5.01954  (22) | 400.043  (76) | *Pbcn* | 0.0162 | 0.01 |

**Table S12** Fractional atomic parameters of delithiated NiNb_2_O_6_ at −10 °C

| ion | site | *x* | *y* | *z* |
| --- | --- | --- | --- | --- |
| Ni^2+^ | 4*c* | 0 | 0.3430(21) | 0.25 |
| Nb^5+^ | 8*d* | 0.1606(85) | 0.1836(77) | 0.7526(24) |
| O^2−^ | 8*d*_1_ | 0.0928(12) | 0.0851(62) | 0.1392(23) |
| O^2−^ | 8*d*_2_ | 0.4271(15) | 0.1269(53) | 0.0675(90) |
| O^2−^ | 8*d*_3_ | 0.7350(36) | 0.1122(75) | 0.9779(10) |

**Table S13** Fractional atomic parameters of lithiated NiNb_2_O_6_ at −10 °C

| ion | site | *x* | *y* | *z* |
| --- | --- | --- | --- | --- |
| Ni^2+^ | 4*c* | 0 | 0.3393(21) | 0.25 |
| Nb^5+^ | 8*d* | 0.1616(95) | 0.1839(86) | 0.7609(11) |
| O^2−^ | 8*d*_1_ | 0.0919(14) | 0.0802(66) | 0.1245(23) |
| O^2−^ | 8*d*_2_ | 0.4266(16) | 0.1262(55) | 0.0725(97) |
| O^2−^ | 8*d*_3_ | 0.7389(36) | 0.1219(73) | 0.1542(28) |

**Table S14** Bond lengths of delithiated and lithiated NiNb_2_O_6_ states at −10 °C.

| bond | delithiated NiNb_2_O_6_ (Å) | lithiated NiNb_2_O_6_ (Å) |
| --- | --- | --- |
| Ni−O (8*d*_1_) | 2.0431(9) | 2.0493(11) |
| Ni−O (8*d*_2_) | 1.8907(7) | 1.8768(8) |
| Nb−O (8*d*_1_) | 2.0433(8) | 2.0907(8) |
| Nb−O (8*d*_2_) | 1.8802(8) | 1.8667(9) |
| Nb−O (8*d*_3_) | 1.8043(7) | 1.8102(9) |

**Table S15** NiO_6_ and NbO_6_ polyhedron-volumes in delithiated and lithiated NiNb_2_O_6_ states at −10 °C

| sample | NiO_6_ (Å^3^) | NbO_6_ (Å^3^) |
| --- | --- | --- |
| delithiated NiNb_2_O_6_ | 10.7223 | 10.9830 |
| lithiated NiNb_2_O_6_ | 11.0163 | 10.8917 |

**Table S16** Lattice parameters of delithiated NiNb_2_O_6_, partially lithiated NiNb_2_O_6_, lithiated NiNb_2_O_6_ after phase transformation, and lithiated NiNb_2_O_6_ at 60 °C

| sample | *a* (Å) | *b* (Å) | *c* (Å) | *V* (Å^3^) | Space group | *R_wp_* | Uiso |
| --- | --- | --- | --- | --- | --- | --- | --- |
| delithiated NiNb_2_O_6_ | 14.04380  (86) | 5.68507  (21) | 5.02643  (16) | 401.287  (33) | *Pbcn* | 0.0151 | 0.01 |
| partially lithiated NiNb_2_O_6_ | 14.04408  (75) | 5.68917  (21) | 5.02945  (16) | 401.830  (49) | *Pbcn* | 0.0137 | 0.01 |
| lithiated NiNb_2_O_6_ after phase transformation | 14.03446  (17) | 5.69336  (49) | 5.03030  (39) | 401.938  (13) | *Pbcn* | 0.0118 | 0.01 |
| lithiated NiNb_2_O_6_ | 14.00864  (27) | 5.68940  (76) | 5.02191  (58) | 400.251  (72) | *Pbcn* | 0.0117 | 0.01 |

**Table S17** Fractional atomic parameters of delithiated NiNb_2_O_6_ at 60 °C

| ion | site | *x* | *y* | *z* |
| --- | --- | --- | --- | --- |
| Ni^2+^ | 4*c* | 0 | 0.3350(20) | 0.25 |
| Nb^5+^ | 8*d* | 0.1581(82) | 0.1813(74) | 0.7710(26) |
| O^2−^ | 8*d*_1_ | 0.0817(13) | 0.0651(51) | 0.1280(54) |
| O^2−^ | 8*d*_2_ | 0.4217(16) | 0.1498(52) | 0.0727(42) |
| O^2−^ | 8*d*_3_ | 0.7580(15) | 0.1226(49) | 0.0917(53) |

**Table S18** Fractional atomic parameters of partially lithiated NiNb_2_O_6_ at 60 °C

| ion | site | *x* | *y* | *z* |
| --- | --- | --- | --- | --- |
| Ni^2+^ | 4*c* | 0 | 0.3352(77) | 0.25 |
| Nb^5+^ | 8*d* | 0.1603(04) | 0.1776(09) | 0.7546(29) |
| O^2−^ | 8*d*_1_ | 0.0754(17) | 0.0514(33) | 0.1345(55) |
| O^2−^ | 8*d*_2_ | 0.4176(20) | 0.1638(91) | 0.0566(06) |
| O^2−^ | 8*d*_3_ | 0.7628(25) | 0.1130(59) | 0.0999(94) |

**Table S19** Fractional atomic parameters of lithiated NiNb_2_O_6_ after phase transformation at 60 °C

| ion | site | *x* | *y* | *z* |
| --- | --- | --- | --- | --- |
| Ni^2+^ | 4*c* | 0 | 0.3250(37) | 0.25 |
| Nb^5+^ | 8*d* | 0.1582(34) | 0.1801(19) | 0.7419(27) |
| O^2−^ | 8*d*_1_ | 0.0808(12) | 0.0422(33) | 0.1395(52) |
| O^2−^ | 8*d*_2_ | 0.4206(22) | 0.1419(89) | 0.0732(16) |
| O^2−^ | 8*d*_3_ | 0.7591(20) | 0.1029(53) | 0.0961(14) |

**Table S20** Fractional atomic parameters of lithiated NiNb_2_O_6_ at 60 °C

| ion | site | *x* | *y* | *z* |
| --- | --- | --- | --- | --- |
| Ni^2+^ | 4*c* | 0 | 0.3247(59) | 0.25 |
| Nb^5+^ | 8*d* | 0.1588(25) | 0.1790(20) | 0.7335(53) |
| O^2−^ | 8*d*_1_ | 0.0870(35) | 0.0677(16) | 0.1314(14) |
| O^2−^ | 8*d*_2_ | 0.4198(39) | 0.1258(16) | 0.0734(13) |
| O^2−^ | 8*d*_3_ | 0.7623(39) | 0.0853(20) | 0.1006(14) |

**Table S21** Bond lengths of delithiated NiNb_2_O_6_, partially lithiated NiNb_2_O_6_, lithiated NiNb_2_O_6_ after phase transformation, and lithiated NiNb_2_O_6_ states at 60 °C

| bond | delithiated NiNb_2_O_6_ (Å) | partially lithiated NiNb_2_O_6_ (Å) | lithiated NiNb_2_O_6_ after phase transformation (Å) | lithiated NiNb_2_O_6_ (Å) |
| --- | --- | --- | --- | --- |
| Ni−O (8*d*_1_) | 2.0220(6) | 2.0362(9) | 2.0456(14) | 2.0230(2) |
| Ni−O (8*d*_2_) | 2.1282(7) | 2.1104(9) | 2.1062(14) | 2.1150(2) |
| Nb−O (8*d*_1_) | 2.0301(6) | 2.0765(7) | 2.1046(15) | 2.0695(3) |
| Nb−O (8*d*_2_) | 1.7745(6) | 1.7530(7) | 1.7237(11) | 1.7404(2) |
| Nb−O (8*d*_3_) | 2.0747(7) | 2.0864(7) | 2.0906(11) | 2.0956(3) |

**Table S22** NiO_6_ and NbO_6_ polyhedron-volumes in delithiated NiNb_2_O_6_, partially lithiated NiNb_2_O_6_, lithiated NiNb_2_O_6_ after phase transformation, and lithiated NiNb_2_O_6_ states at 60 °C

| sample | NiO_6_ (Å^3^) | NbO_6_ (Å^3^) |
| --- | --- | --- |
| delithiated NiNb_2_O_6_ | 11.8765 | 10.4224 |
| partially lithiated NiNb_2_O_6_ | 11.9830 | 10.3433 |
| lithiated NiNb_2_O_6_ after phase transformation | 12.6139 | 10.1045 |
| lithiated NiNb_2_O_6_ | 12.3195 | 10.1537 |

**Supplementary References**

[S1] A.J. Bard, L.R. Faulkner, Electrochemical Methods: Fundamentals and Applications, second ed., Wiley, New York, USA 2001.

[S2] V. Augustyn, J. Come, M.A. Lowe, J.W. Kim, P.L. Taberna et al., High-rate electrochemical energy storage through Li+ intercalation pseudocapacitance, Nat. Mater. **12** (2013) 518-522. <https://doi.org/10.1038/nmat3601>

[S3] R. Xia, K. Zhao, Y. Kuo, L. Zhang, D. M. Cunha et al., Nickel niobate anodes for high rate lithium-ion batteries, Adv. Energy Mater. **12** (2021) 2102972. <https://doi.org/10.1002/aenm.202102972>

[S4] T. Jiang, S. Ma, J. Deng, T. Yuan, C. Lin et al., Partially reduced titanium niobium oxide: A high-performance lithium-storage material in a broad temperature range, Adv. Sci. **9** (2021) 2105119. <https://doi.org/10.1002/advs.202105119>

[S5] X. Lou, Q. Fu, J. Xu, X. Liu, C. Lin et al., GaNb_11_O_29_ nanowebs as high-performance anode materials for lithium-ion batteries, ACS Appl. Nano Mater. **1** (2018) 183-190. <https://doi.org/10.1021/acsanm.7b00091>

[S6] L. Zhao, S. Wang, Y. Dong, W. Quan, F. Han et al., Coarse-grained reduced MoxTi_1−x_Nb_2_O_7+y_ anodes for high-rate lithium-ion batteries, Energy Storage Mater. **34** (2021) 574-581. <https://doi.org/10.1016/j.ensm.2020.10.016>

[S7] P. Jing, K. Liu, L. Soule, J. Wang, T. Li et al., Engineering the architecture and oxygen deficiency of T-Nb_2_O_5_-carbon-graphene composite for high-rate lithium-ion batteries, Nano Energy **89** (2021) 106398. <https://doi.org/10.1016/j.nanoen.2021.106398>

[S8] H. Li, L. Shen, G. Pang, S. Fang, H. Luo et al., TiNb_2_O_7_ nanoparticles assembled into hierarchical microspheres as high-rate capability and long-cycle-life anode materials for lithium ion batteries, Nanoscale **7** (2015) 619-624. <https://doi.org/10.1039/C4NR04847D>

[S9] Q. Fu, J. Hou, R. Lu, C. Lin, Y. Ma et al., Electrospun Ti_2_Nb_10_O_29_ hollow nanofibers as high-performance anode materials for lithium-ion batteries, Mater. Lett. **214** (2018) 60-63. <https://doi.org/10.1016/j.matlet.2017.11.076>

[S10] L. Fei, Y. Xu, X. Wu, Y. Li, P. Xie et al., SBA-15 Confined synthesis of TiNb_2_O_7_ nanoparticles for lithium-ion batteries, Nanoscale **5** (2013) 11102-11107. <https://doi.org/10.1039/c3nr03594h>

[S11] L. Yan, J. Shu, C. Li, X. Cheng, H. Zhu et al., W_3_Nb_14_O_44_ nanowires: Ultrastable lithium storage anode materials for advanced rechargeable batteries, Energy Storage Mater. **16** (2019) 535-544. <https://doi.org/10.1016/j.ensm.2018.09.008>

[S12] L. Xi, H. Wang, S. Yang, R. Ma, Z. Lu et al., Single-crystalline Li_4_Ti_5_O_12_ nanorods and their application in high-rate capability Li_4_Ti_5_O_12_/LiMn_2_O_4_ full cells, J. Power Sources **242** (2013) 222-229. <https://doi.org/10.1016/j.jpowsour.2013.04.020>

[S13] R. Tao, T. Zhang, S. Tan, C. Jafta, C. Li et al., Insight into the fast-rechargeability of a novel Mo_1.5_W_1.5_Nb_14_O_44_ anode material for high-performance lithium-ion batteries, Adv. Energy Mater. **12** (2022) 2200519. <https://doi.org/10.1002/aenm.202200519>

[S14] C. Guo, Z. Liu, K. Han, L. Zhang, X. Ding et al., Nano-sized niobium tungsten oxide anode for advanced fast-charge lithium-ion batteries, Small **18** (2022) 2107365. <https://doi.org/10.1002/smll.202107365>

[S15] X. Lou, Z. Xu, Z. Luo, C. Lin, C. Yang et al., Exploration of Cr_0.2_Fe_0.8_Nb_11_O_29_ as an advanced anode material for lithium-ion batteries of electric vehicles, Electrochim. Acta **245** (2017) 482-488. <https://doi.org/10.1016/j.electacta.2017.05.168>

[S16] Q. Fu, H. Cao, G. Liang, L. Luo, Y. Chen et al., A highly Li+-conductive HfNb_24_O_62_ anode material for superior Li+ storage, Chem. Commun. **56** (2020) 619-622. <https://doi.org/10.1039/C9CC07447C>

[S17] W. Wang, J. Park, V. Nguyen, E. Jin, H. Gu, Hierarchical mesoporous rutile TiO_2_/C composite nanospheres as lithium-ion battery anode materials, Ceram. Int. **42** (2016) 598-606. <https://doi.org/10.1016/j.ceramint.2015.08.153>

[S18] X. Zhu, Q. Fu, L. Tang, C. Lin, J. Xu et al., Mg_2_Nb_34_O_87_ porous microspheres for use in high-energy, safe, fast-charging, and stable lithium-ion batteries, ACS Appl. Mater. Interfaces **10** (2018) 23711-23720. <https://doi.org/10.1021/acsami.8b03997>

[S19] X. Lou, R. Li, X. Zhu, L. Luo, Y. Chen et al., New anode material for lithium-ion batteries: Aluminum niobate (AlNb_11_O_29_), ACS Appl. Mater. Interfaces **11** (2019) 6089-6096. <https://doi.org/10.1021/acsami.8b20246>

[S20] C. Lv, C. Lin, X. Zhao, Rational design and synthesis of nickel niobium oxide with high-rate capability and cycling stability in a wide temperature range, Adv. Energy Mater. **12** (2022) 2102550. <https://doi.org/10.1002/aenm.202102550>

[S21] G. Shao, L. Gan, Y. Ma, H. Li, T. Zhai, Enhancing the performance of Li_3_VO_4_ by combining nanotechnology and surface carbon coating for lithium ion batteries, J. Mater. Chem. A **3** (2015) 11253-11260. <https://doi.org/10.1039/C5TA02094H>

[S22] X. Lan, L. Wang, L. Yu, Y. Li, X. Hu, Synergy of highly reversible ω‑Li_3_V_2_O_5_ anodes and fluorine-containing additive electrolytes promises low-temperature-tolerant Li-ion batteries, ACS Mater. Lett. **3** (2021) 1394-1401. <https://doi.org/10.1021/acsmaterialslett.1c00377>

[S23] G. Liang, X. Jin, C. Huang, L. Luo, Y. Chen et al., Cr^3+^-doped Li_3_VO_4_ for enhanced Li+ storage, Funct. Mater. Lett. **13** (2019) 2050005. <https://doi.org/10.1142/S1793604720500058>

[S24] L. Yang, X. Zhu, X. Li, X. Zhao, K. Pei et al., Conductive copper niobate: superior Li+-storage capability and novel Li+-transport mechanism, Adv. Energy Mater. **9** (2019) 1902174. <https://doi.org/10.1002/aenm.201902174>

[S25] S. Qian, H. Yu, L. Yan, H. Zhu, X. Cheng et al., High-rate long-life pored nanoribbon VNb_9_O_25_ built by interconnected ultrafine nanoparticles as anode for lithium-ion batteries, ACS Appl. Mater. Interfaces **9** (2017) 30608-30616. <https://doi.org/10.1021/acsami.7b07460>

[S26] X. Zhu, J. Xu, Y. Luo, Q. Fu, G. Liang et al., MoNb_12_O_33_ as a new anode material for high-capacity, safe, rapid and durable Li+ storage: structural characteristics, electrochemical properties and working mechanisms, J. Mater. Chem. A **7** (2019) 6522-6532. <https://doi.org/10.1039/C9TA00309F>

[S27] Q. Fu, R. Li, X. Zhu, G. Liang, L. Luo et al., Design, synthesis and lithium-ion storage capability of Al_0.5_Nb_24.5_O_62_, J. Mater. Chem. A **7** (2019) 19862-19871. <https://doi.org/10.1039/C9TA04644E>

[S28] F. Ran, X. Cheng, H. Yu, R. Zheng, T. Liu et al., Nano-structured GeNb_18_O_47_ as novel anode host with superior lithium storage performance, Electrochim. Acta **282** (2018) 634-641. <https://doi.org/10.1016/j.electacta.2018.06.109>

[S29] K.J. Griffith, K.M. Wiaderek, G. Cibin, L.E. Marbella, C.P. Grey, Niobium tungsten oxides for high-rate lithium-ion energy storage, Nature **559** (2018) 556-563. <https://doi.org/10.1038/s41586-018-0347-0>

[S30] C. Lin, G. Wang, S. Lin, J. Li, L. Lu, TiNb_6_O_17_: a new electrode material for lithium-ion batteries, Chem. Commun. **51** (2015) 8970-8973. <https://doi.org/10.1039/C5CC01494H>

[S31] L. Hu, R. Lu, L. Tang, R. Xia, C. Lin et al., TiCr_0.5_Nb_10.5_O_29_/CNTs nanocomposite as an advanced anode material for high-performance Li+-ion storage, J. Alloys Compd. **732** (2018) 116-123. <https://doi.org/10.1016/j.jallcom.2017.10.145>

[S32] W. Ye, H. Yu, X. Cheng, H. Zhu, R. Zheng et al., Highly efficient lithium container based on non-Wadsley-Roth structure Nb_18_W_16_O_93_ nanowires for electrochemical energy storage, Electrochim. Acta **292** (2018) 331-338. <https://doi.org/10.1016/j.electacta.2018.09.169>

[S33] C. Yang, S. Yu, C. Lin, F. Lv, S. Wu et al., C_r0.5_Nb_24.5_O_62_ nanowires with high electronic conductivity for high-rate and long-life lithium-ion storage, ACS Nano **11** (2017) 4217-4224. <https://doi.org/10.1021/acsnano.7b01163>

[S34] L. Hu, C. Lin, C. Wang, C. Yang, J. Li et al., TiNb_2_O_7_ nanorods as a novel anode material for secondary lithium-ion batteries, Funct. Mater. Lett. **9** (2016) 1642004. <https://doi.org/10.1142/S1793604716420042>

[S35] C. Shi, K. Xiang, Y. Zhu, W. Zhou, X. Chen et al., Box-implanted Nb_2_O_5_ nanorods as superior anode materials in lithium ion batteries, Ceram. Int. **43** (2017) 12388-12395. <https://doi.org/10.1016/j.ceramint.2017.06.105>

[S36] W. Wang, Q. Zhang, T. Jiang, S. Li, J. Gao et al., Conductive LaCeNb_6_O_18_ with a very open A-site-cation-deficient perovskite structure: A fast- and stable-charging Li+-storage anode compound in a wide temperature range, Adv. Energy Mater. **12** (2022) 2200656. <https://doi.org/10.1002/aenm.202200656>

[S37] Q. Fu, X. Zhu, R. Li, G. Liang, L. Luo et al., A low-strain V_3_Nb_17_O_50_ anode compound for superior Li+ storage, Energy Storage Mater. **30** (2020) 401-411. <https://doi.org/10.1016/j.ensm.2020.05.012>

[S38] X. Cheng, S. Qian, H. Yua, H. Zhua, Y. Xie et al., BaNb_3.6_O_10_ nanowires with superior electrochemical performance towards ultrafast and highly stable lithium storage, Energy Storage Mater. **16** (2019) 400-410. <https://doi.org/10.1016/j.ensm.2018.06.018>

[S39] J. Gao, L. Yang, C. Huang, G. Liang, Y. Lei et al., Sodium niobate with a large interlayer spacing: A fast-charging, long-life, and low-temperature friendly lithium-storage material, Adv. Sci. **10** (2023) 2300583. <https://doi.org/10.1002/advs.202300583>

[S40] C. Lv, C. Lin, X. Zhao, Rational design and synthesis of nickel niobium oxide with high-rate capability and cycling stability in a wide temperature range, Adv. Energy Mater. **12** (2021) 2102550. <https://doi.org/10.1002/aenm.202102550>

[S41] X. Zhu, H. Cao, R. Li, Q. Fu, G. Liang et al., Zinc niobate materials: crystal structures, energy-storage capabilities and working mechanisms, J. Mater. Chem. A **7** (2019) 25537-25547. <https://doi.org/10.1039/C9TA07818E>

[S42] R. Li, G. Liang, X. Zhu, Q. Fu, Y. Chen et al., Mo_3_Nb_14_O_44_: A new Li+ container for high-performance electrochemical energy storage, Energy Environ. Sci. **4** (2020) 65-71. <https://doi.org/10.1002/eem2.12098>

[S43] S. Patoux, M. Dolle, G. Rousse, C. Masquelier, A reversible lithium intercalation process in a ReO_3_-Type structure PNb_9_O_25_, J. Electrochem. Soc. **149** (2002) A391. <https://doi.org/10.1149/1.1455647>

[S44] S. Li, J. Gao, Y. Ou, W. Wang, Q. Zhang et al., A general strategy to enhance the electrochemical activity and energy density of energy-storage materials through using sintering aids with redox activity: A case study of Mo_4_Nb_26_O_77_, J. Mater. Chem. A **10** (2022) 19953-19962. <https://doi.org/10.1039/D2TA02169B>
